# Three-Dimensional Regulation of Radial Glial Functions by Lis1-Nde1 and Dystrophin Glycoprotein Complexes

**DOI:** 10.1371/journal.pbio.1001172

**Published:** 2011-10-18

**Authors:** Ashley S. Pawlisz, Yuanyi Feng

**Affiliations:** Department of Neurology and Center for Genetic Medicine, Northwestern University Feinberg School of Medicine, Chicago, Illinois, United States of America; University of Cambridge, United Kingdom

## Abstract

Radial glial cells (RGCs) are distinctive neural stem cells with an extraordinary slender bipolar morphology and dual functions as precursors and migration scaffolds for cortical neurons. Here we show a novel mechanism by which the Lis1-Nde1 complex maintains RGC functions through stabilizing the dystrophin/dystroglycan glycoprotein complex (DGC). A direct interaction between Nde1 and utrophin/dystrophin allows for the assembly of a multi-protein complex that links the cytoskeleton to the extracellular matrix of RGCs to stabilize their lateral membrane, cell-cell adhesion, and radial morphology. Lis1-Nde1 mutations destabilized the DGC and resulted in deformed, disjointed RGCs and disrupted basal lamina. Besides impaired RGC self-renewal and neuronal migration arrests, Lis1-Nde1 deficiencies also led to neuronal over-migration. Additional to phenotypic resemblances of Lis1-Nde1 with DGC, strong synergistic interactions were found between Nde1 and dystroglycan in RGCs. As functional insufficiencies of *LIS1*, *NDE1*, and dystroglycan all cause lissencephaly syndromes, our data demonstrated that a three-dimensional regulation of RGC's cytoarchitecture by the Lis1-Nde1-DGC complex determines the number and spatial organization of cortical neurons as well as the size and shape of the cerebral cortex.

## Abstract 2

Lis1-Nde1 integrates cerebral cortical neurogenesis with neuronal migration by stabilizing the basal-lateral surface of radial glial cells.

## Author Summary

The processes of neurogenesis and neuronal migration within the developing cerebral cortex must be tightly orchestrated to enable ordered generation and transportation of neurons to designated cortical layers. The mechanism by which these two processes are integrated remains elusive. Radial glial cells, the major neural stem cells in the developing brain, serve both as progenitors and migration scaffolds for cortical neurons as they migrate. The cortical developmental disease lissencephaly (smooth brain) is a result of defects in neurogenesis and neuronal migration, and is associated with the protein LIS1 and its binding partner NDE1. In this study, we show that several key players in human cerebral cortical development, including LIS1, NDE1, dystrophin, and dystroglycan, form a molecular complex to regulate cortical neurogenesis and neuronal migration in a mouse model. This multi-protein complex is active on the basal-lateral surface of radial glial cells, which is known to provide guidance to migrating neurons. When we depleted NDE1 in mice, dystrophin and dystroglycan were lost from the membrane and radial glial cells were deformed, indicating the importance of the multi-protein complex for proper cell morphology. This effect on morphology resulted in a loss of normal migration and cortical phenotypes similar to lissencephaly. Our findings suggest that genes that regulate the structure and function of the basal-lateral membrane of radial glial cells may integrate the dual functions of these cells and determine the size, shape, and function of the cerebral cortex.

## Introduction

Radial glial cells (RGCs) in the developing cerebral cortex are the most distinctive stem cells, having unique morphology and cytoarchitectural environments. Derived from neuroepithelial cells (NECs) at the onset of neurogenesis, RGCs maintain the apical-basal polarity of NECs but elongate while new neurons are generated and migrate towards the brain margin [1],[2]. With a very narrow apical surface exposed to the ventricular fluid and basal endfeet securely attached to the pial basement membrane (BM), these long and thin cells have vast lateral membranes that are in tight contact with neighboring RGCs through various cell-cell junctions and extracellular matrix (ECM) molecules [3],[4]. Concomitant with increased generation and migration of neurons during mid- to late corticogenesis, the long lateral process of the RGC further extends while newborn neurons migrate through many layers of progenitors and earlier born neurons spanning the entire cortical wall to stop precisely beneath the cortical pial BM [2],[5]–[7]. Although it is conceivable that dynamic controlling the morphology and cell-cell interactions of RGCs is pivotal for their dual functions as progenitors and migration scaffolds for cortical neurons, cell molecular mechanisms that integrate the sophisticated structure, organization, and dual function of RGCs remain largely elusive.

The cerebral cortical developmental disease lissencephaly (smooth brain) is a result of both aberrant cerebral cortical neurogenesis and neuronal migration, and is frequently associated with the haploinsufficiency of *LIS1* [8],[9]. *LIS1* encodes a cytoplasmic protein that achieves multifaceted functions through interacting with cellular proteins of diverse activities. LIS1 appears to be a house-keeping protein as its absence led to peri-implantation lethality, presumably due to the loss of controlled cellular vital functions mediated by its associated microtubules and microtubule-based motors [10]–[13]. We have shown that the central nervous system (CNS) defects caused by LIS1 haploinsufficiency are associated with its binding partner Nde1, a adaptor or scaffold protein that is predominantly detected in neural progenitors but largely devoid in cortical neurons [14],[15]. The Lis1-Nde1 interaction is extremely strong, and a majority of Lis1 protein is predicted to be Nde1 bond based on the high affinity interaction between the two proteins. Besides interacting with Lis1 physically, the CNS and cerebral cortical specific role of Nde1 was further demonstrated by the recent identification of *NDE1* recessive mutations in humans, which showed that loss of *NDE1* function resulted in extreme microcephaly (small brain) and lissencephaly, and that the affected individuals had brains less than 10% of expected size and defective cortical lamination [16],[17]. Therefore, NDE1 is one of the most essential players in determining the size and shape of the cerebral cortex through its integrated regulation of neural progenitor division and neuronal migration.

To understand the fundamental mechanism by which LIS1 and NDE1 control CNS development, we have previously established an allelic series of Lis1 and Nde1 mutant mice, and showed a tight stoichiometric synergistic interaction of the two proteins in cortical neurogenesis and neuronal migration. Double haploinsufficiency of Lis1 and Nde1 (Lis1^+/−^ Nde1^+/−^) phenocopied the Nde1 homozygous loss of function (Nde1^−/−^) in defective cortical neuronal progenitor mitosis, which led to a small but grossly laminated cerebral cortex. Further reducing the dosage of Lis1-Nde1 complex by Lis1 heterozygous and Nde1 homozygous double mutations resulted in mice that resembled *NDE1* recessive mutations in humans. The cerebral cortex of these mutant mice was less than 20% of the normal size with disorganized and inverted neuronal layers, whereas most of the tissues and organs outside of the CNS remained grossly normal in both size and structure [18]. The dramatic impairment of neural progenitor self-renewal in the Lis1^+/−^ Nde1^−/−^ mutant sharply correlated with the initial morphological transition of NECs to RGCs. Despite remarkably reduced ratio of symmetrical proliferative over asymmetrical neurogenic divisions of RGCs, very subtle defects were detected in NECs and progenitors of the subventricular zone (SVZ) in the mutant, indicating not only the CNS-specific but also the RGC-specific requirement of the Lis1-Nde1 complex.

Both Lis1 and Nde1 are scaffold proteins of which subcellular localizations may be dynamically regulated under various cellular physiological conditions. As scaffold proteins, both Lis1 and Nde1 conduct functions through protein-protein interactions that mediate the formation of molecular complexes required for cell signaling and/or cell mechanics. The strong physical and dosage-dependent genetic interaction between Lis1 and Nde1 indicated that the two proteins together establish or stabilize multi-molecular complexes in the RGC, but the molecular complexes through which Lis1 and Nde1 regulate the unique features of RGCs are not well defined. Up to now the understanding of the RGC-specific requirement of the Lis1-Nde1 is limited to their association to the mitotic apparatus. Both Lis1 and Nde1 have been functionally implicated in microtubule organization, dynein motor force production, centrosome duplication, and mitotic spindle assembly; both have been shown to play roles in maintaining the self-renewing symmetric division of RGCs through regulating mitotic spindle orientations [13]–[15]. Nonetheless, the mechanism by which the Lis1-Nde1 complex regulates spindle orientation in RGCs is not fully understood. Although previous studies have shown that Ndel1, the mammalian paralogue of Nde1, mediates the cortical capture of astral microtubules and anchors the dynein motor complex to the cell cortex [13], it is unclear how Ndel1 or Nde1 is recruited to the cell surface. Recent in vitro analysis suggests that LIS1 and Nde1/Ndel1 play a role in modulating dynein motor force generation to transport nuclei, centrosomes, or chromosomes [19], but it is unclear how LIS1 and Nde1 deficiencies impair the dynein motor function specifically in RGCs but not other somatic cells in vivo. Despite deficiencies in cortical neurogenesis, lissencephaly is primarily known as a cortical neuronal migration disease, and the neuronal migration defect of Lis1 heterozygous mutation could be significantly enhanced by Nde1 mutations [18]. How does the Lis1-Nde1 complex regulate cortical neuronal migration? Does the Lis1-Nde1 complex regulate the motility of cortical neurons directly or primarily through non-cell-autonomous regulations of the RGC scaffold? As cortical neurogenesis and neuronal migration are precisely orchestrated, is it possible that the Lis1-Nde1 complex regulates these two important developmental events through a shared molecular mechanism? In order to answer these questions and understand the RGC-specific function of Lis1 and Nde1, we have set out to search for the Lis1-Nde1 regulated molecular complexes and mechanisms in RGC that may be commonly important for neurogenesis and neuronal migration. Our previous analysis of the Lis1^+/−^ Nde1^−/−^ mutant suggested that the severe neurogenesis and neuronal migration abnormalities were tightly correlated with striking alterations in the radial morphology and loss of basal-lateral adhesions of the mutant RGCs [18], suggesting a previously unrecognized mechanism by which the Lis1-Nde1 complex regulates the basal-lateral cell surface mechanics of the RGC [18]. In this study, we describe the new finding that Lis1-Nde1 interacts with the dystrophin/dystroglycan glycoprotein complex (DGC). The Lis1-Nde1-DGC complex allows for the formation of a bridge between Lis1-Nde1 regulated microtubule associated structures with DGC bound actin cytoskeleton and ECM. This complex plays an essential role in maintaining the integrity of RGC's lateral membrane surface, anchoring the astral microtubules to the cell cortex and promoting RGC-RGC or RGC-neuron interactions. This newly discovered mechanism of Lis1-Nde1 appears to be responsible for establishing the radial morphology and the cytoarchitecture of RGCs, which are essential for integrating the dual-function of RGCs to assure both normal cortical neurogenesis and neuronal migration. More interestingly, functional deficiencies of DGC have also been known to associate with lissencephaly [20]. The Lis1-Nde1 double deficient mouse mutant described in this study presented the pathology of NDE1, LIS1, and DGC deficient patients. Our findings therefore provide a new framework for understanding the complex pathogenesis of developmental brain malformation diseases as well as cell molecular mechanisms governing the developmental and evolutionary formation of the human cerebral cortex.

## Results

### The Plasma Membrane Association of Nde1

**Figure 1:**
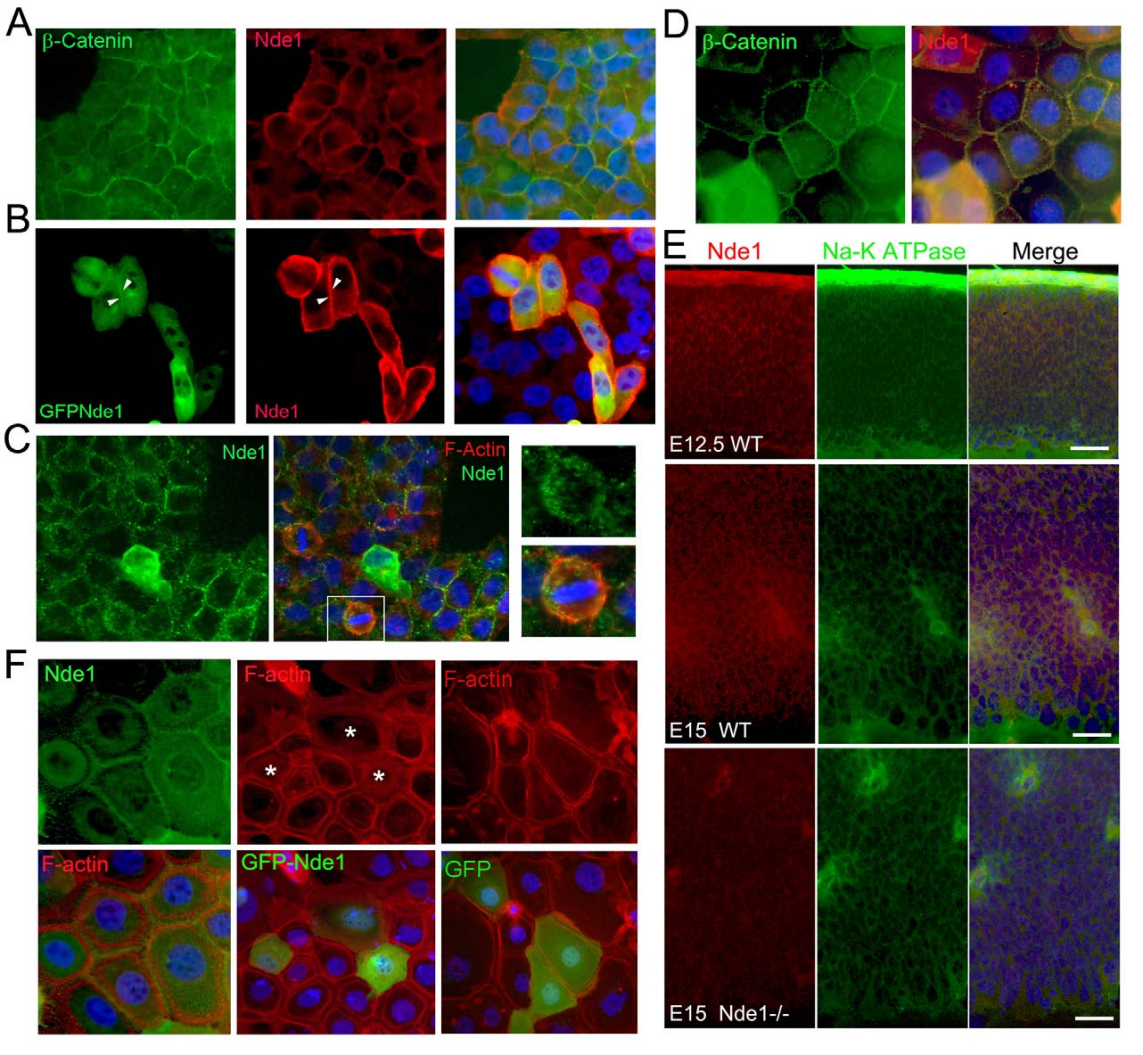
Nde1's association with the plasma membrane cytoskeleton. (A) Using fixation conditions that stabilize the plasma membrane, double immunofluorescence staining with antibodies to Nde1 (red) and the cell-cell junction protein β-catenin (green) demonstrate co-localization. (B) A fraction of overexpressed GFP-Nde1 (green), recognized by the Nde1 antibody (red), was observed at the cell-cell junctions (arrow heads). (C) Nde1 (green) was also seen at the cell cortex in metaphase cells, where it partially co-localized with cell cortical F-Actin (red). (D) A better co-localization of Nde1 with β-catenin was also seen in skin epithelial derived SCC9 cells. (E) Immunohistological analysis identified a pool of Nde1 (red) that co-localizes with Na-K ATPase α-subunit on the basal-lateral surface of radial glial progenitors in the developing cerebral cortex. Tissue sections of Nde1−/− brains were used as negative controls for antibody specificity. Bars: 50 µm. (F) Overexpressed GFP-Nde1 (green) in SCC9 cells destabilized the cortical F-actin (red) cables at the cell-cell junction. Nde1 denotes endogenous Nde1, and GFP and GFP-Nde1 denote overexpressed recombinant proteins. Cells with destabilized cortical actin are indicated by asterisks.

To explore the molecular mechanism by which the Lis1-Nde1 complex stabilizes the cell morphology and cell-cell adhesions of RGCs, we carefully re-examined the subcellular localization of Nde1 and identified a new cell surface associated pool of Nde1. Nde1 was known to localize at the centrosome as well as key sites for mitotic spindle assembly to confer a critical role in regulating the organization of both interphase and mitotic microtubules [14],[15]. However, under fixation conditions that protect the plasma membrane, a significant fraction of Nde1 was detected at the cell surface as revealed by immunofluorescence staining with antibodies specific to Nde1. In epithelial derived Hela and SCC9 cells, Nde1 immunoreactivity co-localizes with β-catenin at the cell-cell junctions, suggesting an association of Nde1 with the plasma membrane (Figure 1A–D). In both interphase and mitotic cells, the cell surface-bound Nde1 localized with or in the vicinity of cell cortical actin (Figure 1C,F). In the ventricular zone of mouse developing cerebral cortex, immunohistological signals of Nde1 showed a significant overlap with that of the Na-K ATPase, a housekeeping protein on the basal-lateral membrane of RGCs (Figure 1E). A substantial amount of recombinant GFP-Nde1 could be observed at the surface of Hela cells (Figure 1B, arrows). The overexpressed GFP-Nde1 also disrupted cortical actin cables in SCC9 cells (Figure 1F). These data are consistent with the recent report that NDE1 and its paralog NDEL1 are enriched in membrane-bound cell fractions [21], and they together demonstrate that Nde1 has a previously unrecognized role as part of the cell surface membrane cytoskeleton. The plasma membrane associated pool of Nde1 might be essential for maintaining the basal-lateral membrane stability and/or adhesion of RGCs during cerebral cortical development.

### Nde1 Interacts with Membrane Skeleton Proteins Utrophin and Dystrophin

To further reveal the mechanism by which Nde1 regulates the basal-lateral surface mechanics of the RGC, we searched for Nde1 associated proteins at the cell surface and identified that Nde1 interacted directly with Utrophin and Dystrophin. Through screening a mouse E9.5–10.5 whole embryo yeast two-hybrid library [22], we pulled out multiple clones that encode the C-terminus of Utrophin (Utrn). Utrn is a widely expressed and functionally interchangeable homologue of Dystrophin (Dmd), the protein absent in patients with Duchenne and reduced in Becker muscular dystrophies [23],[24]. Both Utrn and Dmd are large cytoplasmic proteins required for structural stability of the sarcolemma of muscle cells by connecting the actin cytoskeleton to extracellular matrix (ECM) [25],[26].

**Figure 2:**
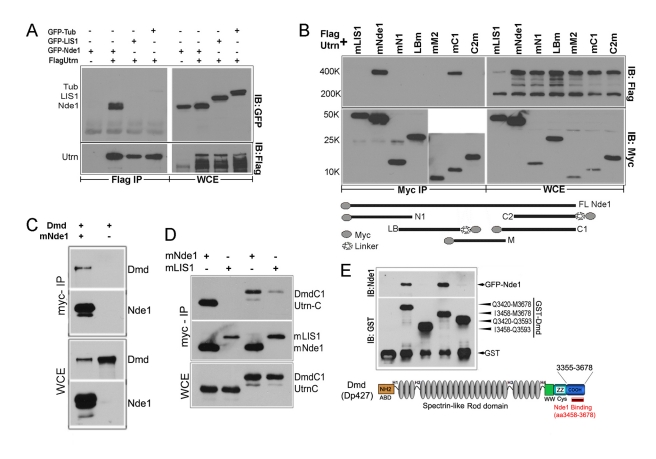
Interaction of Nde1 with Utrn and Dmd. (A) Full-length Flag-Utrn was co-expressed with GFP tagged-Nde1, LIS1, and Tubulin in 293T cells. Immunoprecipitation was performed with the anti-Flag antibody, and immunoblots were probed by an anti-EGFP antibody. Only GFP-Nde1 was detected in the Flag-Utrn immune-complex. (B) Flag-Utrn was co-expressed with myc-tagged LIS1, myc-tagged full-length, and truncated Nde1 as depicted. (N1, aa1–93; LB, aa88–156; M, aa144–221; C1, aa232–344; C2, aa278–341). Immunoprecipitation was performed with the anti-myc antibody 9E10, and immunoblots were probed with an anti-Flag antibody. LB and C2 were tagged by the myc 9E10 epitope at the C-terminus via a random linker in the pcDNA3.1, which gave them an appearance of higher molecular weights. * Due to the extreme size difference between Utrn (400 kDa) and some Nde1 truncation constructs (<20 kDa), myc immunoprecipitates in (C) were split into three identical parts and analyzed by different electrophoresis and transfer conditions on separate immunoblots. (C) Full-length Flag-Dmd was co-expressed with myc-Nde1 in 293T cells. It was specifically detected in the myc-Nde1 immunoprecipitates by immunoblotting with a Dmd antibody. (D) Myc-tagged Nde1 and LIS1 were co-expressed with EGFP-tagged C-terminal fragments of Utrn and Dmd, respectively, and immunoprecipitated by the 9E10 anti-myc antibody. Both UtrnC(aa 3173–3311) and DmdC1(aa 3458–3678) were specifically detected in the immunoprecipitates of myc-Nde1. (E) GST-Dmd C-terminal fusion proteins were expressed and purified from bacteria on glutathione-agarose. These purified proteins on glutathione beads were further incubated with protein extractions from EGFP-Nde1 transfected 293T cells. The binding of Nde1 to GST-Dmd C-terminal fragments were detected by immunoblottig with an anti-GFP antibody. The structure and amino acid residuals of the Nde1 binding domain of mouse full-length dystrophin (GenBank NM004006) were depicted based on the alignment with DMD (GenBank NM004010).

The interaction of Nde1 with Utrn was confirmed by co-immunoprecipitation analyses. When Flag tagged full-length Utrn was co-expressed with Nde1, not only was Nde1 specifically detected in the immunoprecipitates of Flag-Utrn (Figure 2A), but also a substantial amount of Flag-Utrn was found in the Nde1 immunocomplex (Figure 2B). As a 400 kDa protein, the recombinant full-length Utrn expressed in cell culture was relatively sensitive to proteolysis, but its level could be increased by Nde1 co-transfection (Figure S1), suggesting that Nde1 stabilizes Utrn. The Utrn yeast two-hybrid clone that contains the Nde1 binding domain encodes amino acids 3173 to 3311 of mouse full-length Utrn. This region is adjacent to the ZZ domain near the carboxyl terminus [27] and shares over 85% homology with Dmd. We therefore tested the interaction of Nde1 with Dmd through co-immunoprecipitation. We found that similar to Utrn, both full-length Dmd and its C-terminus could be specifically detected in Nde1 immunoprecipitates (Figure 2C,D). A direct interaction between the C-terminus of Dmd and Nde1 was further examined by using bacteria expressed GST fusion proteins, which demonstrated that 220 residues of Dmd (I3458-M3678) in the C-terminal coiled-coil domain were sufficient for its specific interaction with Nde1 (Figure 2E). The domain by which Nde1 interacts with Utrn/Dmd was identified to be within the C-terminus 112 amino acids through examining several Nde1 truncation constructs for their ability to co-immunoprecipitate Utrn (Figure 2B). This Utrn/Dmd interaction domain is missing in two of the *NDE1* alleles that cause micro-lissencephaly [16],[17]; it is evolutionarily less conserved [14] and does not overlap with the recently identified Dynein interaction domain [28], as well as the previously defined Nde1 dimerization and LIS1 binding domains in the conserved N-terminal coiled-coil segment. Thus, this suggests that Nde1 could bind Lis1 and Utrn or Dmd simultaneously underneath the plasma membrane in evolutionarily more advanced cells. Since Lis1 has also been shown to interact with the plasma membrane reelin receptor VLDLR [29], these data suggest that Nde1 and Lis1 act together to maintain the morphology, surface stability, and lateral adhesion of RGCs through regulating Utrn or Dmd and their associated protein complexes and structures.

### Lis1-Nde1 Deficiency Destabilizes the Dystrophin-Glycoprotein Complex in RGCs

**Figure 3:**
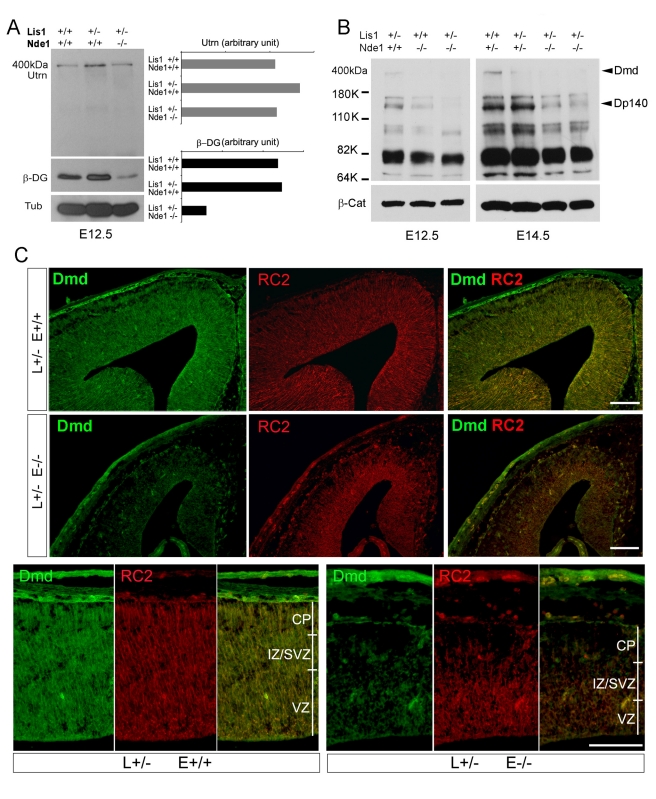
Destabilization of dystrophin and β-DG by Lis1-Nde1 deficiency. (A) Total protein extracts from cerebral cortices of E12.5 embryos were examined by immunoblotting. In comparison to Utrn and Tubulin, a significant decrease in the 43 kda β-DG protein was found in the Lis1^+/−^Nde1^−/−^ mutant. (B) Immunoblotting analyses of total protein extracts from the developing cerebral cortex at E12.5 and E14.5 both revealed decreased signals of an anti-dystrophin antibody (MANDRA1, against aa 3200–3684 of DMD) in the Lis1^+/−^Nde1^−/−^ mutant. Besides full-length Dmd, the most significant loss was the Dp140 isoform of dystrophin, of which deletions underlie the intellectual impairment in up to 30% cases of Duchenne's muscular dystrophy. (C) Immunohistological analysis with antibodies to Dmd (green) and radial glial cell marker RC2 (red) showed reduced Dmd along the basal processes of Lis1^+/−^Nde1^−/−^ RGC at E13.5; bars: 50 µm. CP, cortical plate; IZ, intermediate zone; SVZ, subventricular zone; VZ, ventricular zone; L, Lis1; E, Nde1.

Dmd and Utrn are known to function with the membrane associated receptor dystroglycan (DG/Dag1) and form the dystrophin-associated glycoprotein complex (DGC) [26],[30]. Dag1/DG is translated from a single transcript but is cleaved into α and β-DG post-translationally. While the β-DG is a membrane-spanning molecule that interacts directly with the ZZ domain of Utrn and Dmd, α-DG becomes glycosylated and binds such ECM proteins as laminin [31]. Although the DGC has been better understood by its role in maintaining the plasma membrane stability of muscle fibers, Duchenne muscular dystrophy (DMD) is frequently associated with a spectrum of developmental cognitive behavior disabilities and mental retardation. Morphogenetic abnormalities have been found in DMD brain pathological specimens, suggesting an essential requirement of the DGC in the developing brain [32]. To explore the physiological significance of Nde1-Utrn/Dmd interaction in brain development, we investigated how altered Nde1 may affect the DGC by examining Utrn, Dmd, and dystroglycan levels and distributions in Lis1-Nde1 mutant's cerebral cortex. Although a decrease in the 400 kDa Utrn was undetectable, significant reductions of Dmd and β-DG proteins were detected in the Lis1^+/−^ Nde1^−/−^ cortex from E12.5 to E14.5 (Figure 3A,B, Figure S2). Interestingly, besides the 427 kDa full-length protein, the most pronounced loss of Dmd was seen in the 140 kDa isoform (Dp140), which is predominantly expressed in the developing brain and associates with the cognitive impairment of dystrophinopathies [33]. In contrast to the wide expression of Utrn, the expression of Dmd in the developing cerebral cortex is more confined in the ventricular zone neural progenitors (www.genepaint.org). Using several Dmd monoclonal antibodies, immunohistological analyses consistently indicated the presence of Dmd along the lateral surface of RGC during early cortical development. The Lis1^+/−^ Nde1^−/−^ RGCs were previously found to be severely deformed or truncated basal-laterally with reduced RGC-RGC adhesions [18]. Moreover, in comparison to general RGC markers such as RC2, we observed that the amount of Dmd in these deformed RGCs was significantly reduced in the ventricular zone of the Lis1^+/−^ Nde1^−/−^ cortex (Figure 3C). These experimental evidences support a role of Dmd in regulating the lateral surface integrity and adhesions of RGCs through interacting with the Lis1-Nde1 complex.

**Figure 4:**
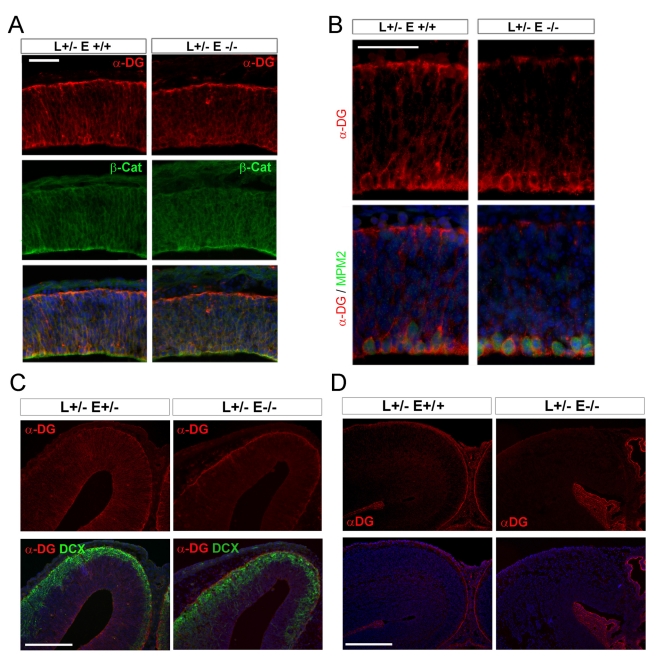
Reduced glycol-α-DG caused by Lis1-Nde1 deficiency. (A) Immunohistological analysis with IIH6 monoclonal antibody (against the laminin binding domain of glycosylated α-DG) showed strong presence of glycol-α-DG (Red) in the cortical ventricular zone (VZ) progenitors of both control and Nde1^−/−^Lis1^+/−^ mutant brains at E11.5; bar: 100 µm. (B) At E12.5, while the basally associated glycol-α-DG starts to decline in Nde1^−/−^Lis1^+/−^ RGCs, glycol-α-DG signals surrounding the M-phase progenitors (identified by MPM2 monoclonal antibody in green) along the ventricle remained detectable; bar: 50 µm. (C) Glycol-α-DG (red) in the Lis1^+/−^ Nde1^−/−^ neocortical ventricular zone was further reduced significantly compared to the control cortex at E13.5, when only RGC basal endfeet associated glyco-α-DG remained to be seen. Bar: 100 µm. (D) By E15.5, while glycol-α-DG (red) was restricted to the radial glial basal endfeet in the control cortex, it became undetectable in the Nde1^−/−^Lis1^+/−^ cortex; bar: 100 µm. Sections were routinely co-stained with Hoechst in blue to reveal the tissue structure and cell organization. L, Lis1; E, Nde1.

Correlated with reduced β-DG, Lis1^+/−^ Nde1^−/−^ mutation also resulted in defective glycol-α-DG in the developing cerebral cortex. IIH6 and VIA4-1 are monoclonal antibodies to the glycosylated species of α-DG [25],[34],[35]. Although α-DG was known to localize to the glial endfeet, both α-DG antibodies reacted robustly to the ventricular zone neural progenitors in early cortical development from E10.5 to E13.5 (Figure 4A, Figures S3 and S4). Intense IIh6 immune-signals of glycol-α-DG were observed at the basal endfeet as well as along the entire basal lateral surface of NECs and RGCs. Enhanced glycol-α-DG immunosignals were also associated with the apically retracted cell body of metaphase progenitors identified by the MPM2 mitotic phospho-protein monoclonal antibody (Figure 4B). This spatial distribution of glycol-α-DG is well in line with a role in mediating the lateral adhesion of RGCs and serving as part of the cell cortical cues that allow precise control of mitotic spindle orientation by Nde1 [36].

Further supporting a role of the Lis1-Nde1 complex in controlling RGC functions by stabilizing the DGC, we found that glycol-α-DG's level and distribution were altered by Lis1-Nde1 deficiency. Reduced glycol-α-DG in the neocortical ventricular zone of Lis1^+/−^Nde1^−/−^ mutant only became evident after neurogenesis commences and NECs transform into RGCs (Figure 4B,C), suggesting the link between Lis1-Nde1 and DGC is RGC specific. Glycol-α-DG localized along the lateral surface of RGCs appeared to be most sensitive to Nde1-Lis1 deficiency and showed decreased levels in the Lis1^+/−^Nde1^−/−^ cortex at E12.5 (Figure 4B, Figure S4). By E13.5, glycol-α-DG remained strongly associated with the lateral membrane of normal RGCs, whereas it could only be detected in the endfeet of mutant RGCs (Figure 4C). After E15.5, glycol-α-DG was predominantly restricted to the basal endfeet of normal RGCs, it became undetectable in the Lis1^+/−^ Nde1^−/−^ cortex (Figure 4D). These data demonstrated that glycol-α-DG is distributed along the lateral surface of RGCs during early corticogenesis when symmetric divisions were dominant and only became restricted to the basal endfeet when symmetrical divisions were taken over by asymmetrical divisions after E15.5. They together suggested that DGC is required for RGC's apical-lateral membrane stability during their early proliferation phase. Thus, the precocious loss of DGC due to Lis1^+/−^Nde1^−/−^ mutation might underlie the reduced cell-cell adhesion, altered mitotic orientation, and failed self-renewal of the mutant RGCs at E11–13 [18]. Although apoptosis of nascent cortical neurons, which peaked around E12.5, was one of the major outcomes of precocious and abnormal neurogenesis caused by the Lis1^+/−^Nde1^−/−^ mutation [18], loss of lateral adhesion and DGC proteins were observed in the VZ of the mutant cortex where apoptosis was devoid. Moreover, programmed cell death decreased after E12.5 and became almost undetectable in the neocortex of the mutant after E14.5 (Figure S5). In contrast, reduced DGC proteins in RGCs peaked when apoptosis was largely absent in the mutant. The spatiotemporal and cell type discorrelation between apoptosis and DGC destabilization made it highly unlikely that decreased DGC proteins in RGCs was a result from apoptosis of mutant neurons.

Dystroglycan has previously been known by its role in stabilizing the radial glial endfeet. However, recent evidence has shown that it performs broad functions in cerebral cortical development. While discontinuous basal lamina was the major phenotype of inactivating dystroglycan after E14.5 by the GFAP-Cre mediated conditional mouse mutation [37], more recent analyses of mice with earlier and broader inactivation of Dag1 resulted in pelotropic defects including microcephaly, disorganized cortical layering, and neuronal overmigration [38]. In contrast, cortical histogenesis was preserved in mice with neuron-specific deletion of dystroglycan [39], supporting the notion that the DGC is essential for specifically assisting the function of the Lis1-Nde1 complex in RGCs rather than in cortical neurons.

### Basal Lamina Defect Caused by RGC Impairments

**Figure 5:**
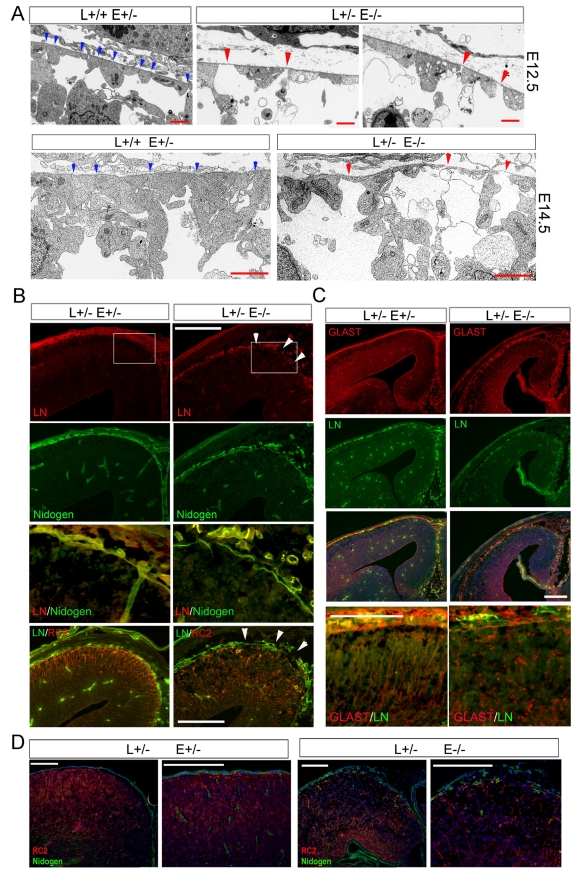
Aberrant RGC structure led to BM destabilization. (A) Electron micrographs of the pial/meningal interphase and glial limitans normal (Lis1^+/+^ Nde1^+/−^) and Lis1^+/−^ Nde1^−/−^ brains at E12.5 and E14.5, respectively. Blue arrowheads indicate electron dense lateral cell-cell contacts between radial glial endfeet. Such structures were largely absent in the Lis1^+/−^ Nde1^−/−^ mutant. Red arrowheads indicate gaps between the radial glial endfeet of the Lis1^+/−^ Nde1^−/−^ mutant. Bars: 2 µm. (B) Cortical sections of E13.5 embryos double immunostained with antibodies to ECM proteins laminin (Red) and nidogen (green). While discontinuous laminin (LN) distribution along the BM was visible in the Lis1^+/−^ Nde1^−/−^ mutant (arrowheads), nidogen immunoreactivity stayed relatively intact, resulting in reduced co-immunolabeling of LN and nidogen. Bars: 50 µm. (C) Immnohistological analyses with radial glial markers GLAST (c) and RC2 (b, d) (red) revealed striking basal lateral structural truncation and deformation in the Lis1^+/−^ Nde1^−/−^ RGCs. The most drastic RGC morphology defect was associated with disrupted laminin integrity (Green) in the medial cortical region (b, c). Bars: 50 µm. (D) Double immunohistological staining with RC2 (red) and nidogen (green) antibodies on cortical sections of E15.5 embryos, showing the loss of BM integrity with the destabilization of nidogen in the Lis1^+/−^ Nde1^−/−^ mutant. Bars: 50 µm. Sections were routinely co-stained with Hoechst in blue to reveal the tissue structure and cell organization. L, Lis1; E, Nde1.

To further delineate the consequence of Lis1-Nde1-DGC destabilization, we examined structural defects of Lis1-Nde1 deficient RGCs by electron microscopy. Aside from the severe loss of apical-lateral RGC-RGC contacts in the ventricular zone [18], we found that the Lis1^+/−^Nde1^−/−^ RGCs disjoined across the entire basal lateral surface. At the basal-most end beneath the cortical pia, although the endfeet of Lis1^+/−^ Nde1^−/−^ RGCs appeared to make contact to the pial-meningeal basement membrane (BM), they showed little interaction with neighboring RGC endfeet. The electron-dense cell-cell junctions that link the endfeet of normal RGCs were rarely seen in the Lis1^+/−^ Nde1^−/−^ mutant. Instead, large gaps were frequently observed between the endfeet of adjacent mutant RGCs (Figure 5A).

Strikingly aberrant morphology and disintegrated cell-cell or cell-BM interaction were also revealed in the basal processes of Lis1^+/−^ Nde1^−/−^ RGCs by immunohistological studies. The cortical BM forms between the endfeet of RGCs (glia limitans) and meningeal fibroblasts, which are composed of extracellular matrix (ECM) molecules including laminin, collagen, perlecan, and nidogen. From E13.5 to 15.5, a significant fraction of Lis1^+/−^Nde1^−/−^ RGCs showed loss of anchorage to the BM as indicated by reduced co-immunostaining of RGC markers RC2 and GLAST with BM associated laminin and nidogen (Figure 5B–D).

Fragmentation of the cortical basal lamina, as evidenced by disorganized and discontinuous laminin and nidogen, was often observed. The severity of RGC's basal lateral defects often shows a medial-lateral gradient in which intense basal lateral thinning, shortening, detachments, and BM breaches of RGCs were detected more frequently in medial regions (Figure 5B–D). As deformed RGC basal processes and disrupted continuous distribution of ECM proteins were highly correlative, RGC defects could be primarily responsible for BM disintegration in the mutant. Moreover, destruction of laminin's continuity in the glial lamitan occurred preferentially to that of nidogen. Laminin fragmentation could be detected as early as E13.5 (Figure 5B), along with the loss of immunoreactivity of the IIH6 antibody (directed against a laminin-binding α-DG glycopeptides [35]). While BM associated nidogen was still largely intact at E13.5, loss of its structural integrity only became evident after E15.5 (Figure 5B,D). As laminin, but not nidogen, is a direct ligand of glycol-α-DG [40], this further suggested that the impaired BM resulted from the destabilization of DGC by Lis1-Nde1 deficiency in RGCs.

### The Type II Lissencephaly-Like Phenotype Caused by Lis1-Nde1 Deficiency

**Figure 6:**
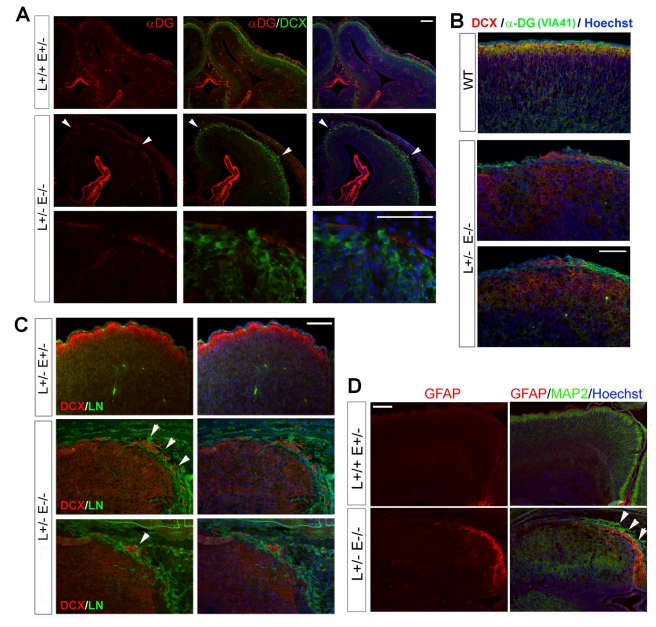
The cobblestone lissencephaly-like phenotypes and the co-existence of neuronal under- and over-migration in Lis1^+/−^Nde1^−/−^ mutants. (A, B) Cortical sections of E13.5 (a) and E15.5 (b) embryos were double immunostained with antibodies to glycol-α-DG (IIH6 in red or VIA41 in green) and a young neuron marker DCX (in green or red as indicated). Regional neuronal ectopia was observed with disrupted α-DG along the BM in the Lis1^+/−^ Nde1^−/−^ mutant. Bars: 100 µm. (C) Immuno-histological analysis of E18.5 Lis1^+/−^Nde1^−/−^ brains revealed widespread cobblestone lissencephaly-like phenotype characterized by ectopically over-migrated CP neurons (marked by DCX in red) through the basement membrane (marked by laminin in green) into the marginal zone/subarachnoid space. Bar: 100 µm. (D) Double immunohistological analyses of E18.5 brain sections with GFAP (red) and MAP2 (green) antibodies both demonstrated increased ectopic neurons and astracytes in the medial cortical region of the Nde1^−/−^Lis1^+/−^ mutant (arrows). Bar: 100 µm. Sections were routinely co-stained with Hoechst in blue to reveal the tissue structure and cell organization. L, Lis1; E, Nde1.

Losing the integrity of cortical basal lamina is known to be associated with a class of cerebral cortical developmental disorders that are collectively classified as type-II lissencephaly [41],[42]. In addition to the smooth cerebral surface and disorganized neuronal layers, this type of lissencephaly is pathologically defined by the “cobblestone” (ectopia) on the surface of the brain due to “overmigration” of cortical neurons into the subarachnoid space. The disorder has been known in several recessive human genetic syndromes that primarily affect the muscle, eye, and brain [43]. The causative genes of these disorders have been found to encode a group of glycosyltransferases that catalyze O-linked glycosylation, and dystroglycan is by far their best characterized substrate in both muscle and brain [43]–[46]. Despite severe neuronal migration arrest beneath the un-splitted preplate [18], we found that disruption of DGC by the Lis1^+/−^ Nde1^−/−^ mutation also induced type-II lissencephaly-like defects with neuronal “overmigration.” Besides densely packed Cajal-Retzius (C-R) cells in the normally cell sparse marginal zone (MZ) [18], neuronal ectopia outside of the glia limitan were frequently observed in the mutant cortex. Although the overmigration of cortical neurons was regional, it always coexisted with breaches of α-DG, suggesting that the glycol-α-DG deficiency is responsible for the neuronal ectopia (Figure 6A,B). Shortly before the death of the mutant at birth, widely spread over-migrated cortical neurons could be seen in medial neocortical regions (Figure 6C). At the same time, dramatically increased GFAP-positive glial astrocytes obliterated the medial cortical subarachnoid space together with dysplastic neuronal ectopia in the mutant (Figure 6D). Such mixed glial and neuronal heterotopias are also reminiscent of those observed in postmortem cases of type-II lissencephaly, as well as mice with Dag1 mutations [37],[47]. Coinciding with the brain developmental defects, the Lis1^+/−^ Nde1^−/−^ mutant also displayed severe muscle atrophy and fibrosis (Figure S6), supporting an essential function of the Lis1-Nde1-DGC complex in muscle development and function.

### Synergistic Regulation of Cortical Neuronal Migration by Nde1 and Dag1

**Figure 7:**
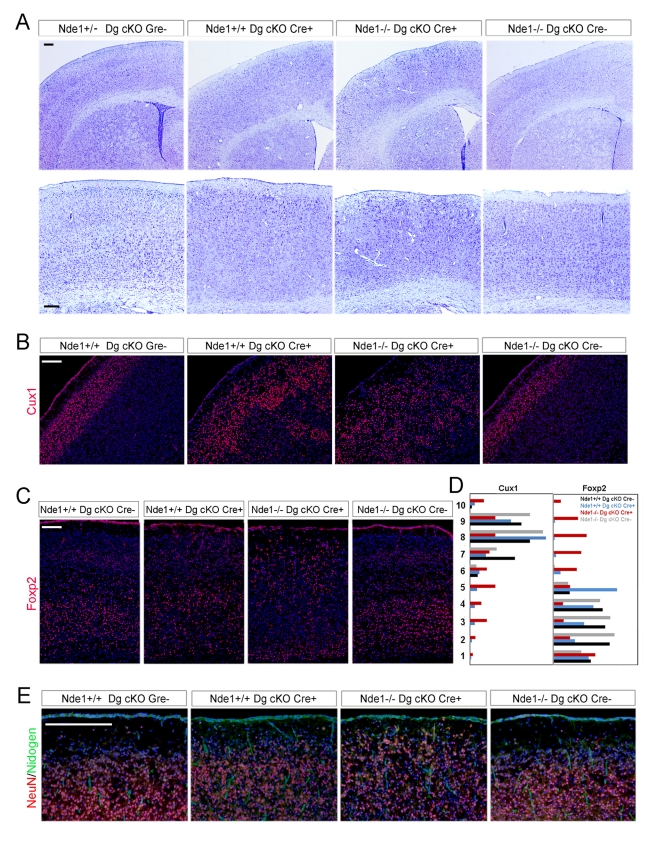
Synergistic interaction of Nde1 with Dag1. (A) Histological analysis of Nde1, Dag1 double mutants. Brain sections of 4-mo-old adult mice were stained with Cresyl violet to view cortical neuronal lamination of indicted mutants. More than 4 litters were analyzed; the Nde1 Dag1 double deficient brains showed significantly more severe disorganization of cortical neurons; the penetrance of synergistic enhancement of Nde1 and Dag1 signal mutant phenotypes by the double mutant was found to be 100%; representative pictures of spatially matched cortical sections of one set of the littermates were shown. Bars: 100 µm. (B) Immunohistological analysis of cortical superficial layer II/III neurons with the Cux1 antibody (red) on early postnatal (P3.5) spatially matched coronal brain sections of indicated genotype. Six litters were analyzed; the synthetic neuronal migration defect of Nde1 and Dag1 double mutations was found with a 100% penetrance; representative pictures of spatially matched cortical sections of one set of the littermates were shown. Bar: 100 µm. (C) Immunohistological analysis of cortical deep layer V/VI neurons with the FoxP2 antibody (red) on early postnatal (P3.5) spatially matched coronal brain sections of indicated genotype. Six litters were analyzed; representative pictures of spatially matched cortical sections of one set of the littermates were shown. Bar: 100 µm. (D) Cortical distribution of Cux1+ and Foxp1+ neurons of indicated mutants. The neocortex of each cortical section was divided equally into 10 layers between the lateral ventricle and the pial surface. Layer 1 is next to the ventricle and layer 10 is immediately beneath the pial surface. About 1,000 Cux1+ neurons or Foxp2+ neurons from 4–5 sets of littermates were analyzed. The fractions (%) of Cux1+ or Foxp2+ neurons in each layer were plotted to indicate the relative distance of neuronal migration from the ventricular surface. (E) Immunohistological analysis of ectopically over-migrated neurons in the MZ. Mature cortical neurons were immunostained by NeuN antibody (red). The cortical pial surface was highlighted by Nidogen immunoreactivity (green) and all sections were stained with Hoechst to view general cell distribution. Bar: 100 µm.

In addition to the phenotypic resemblance of Lis1-Nde1 and DGC mutations, we also found a strong genetic interaction between Nde1 and Dag1. Mice lacking Nde1 showed severe cortical neurogenic defect but moderate neuronal migration delay, resulting in a small but grossly laminated cortex [15]. However, inactivation of Dag1 in Nde1^−/−^ mice led to deteriorating defects in both cortical neurogenesis and neuronal migration. Similar to the previously reported conditional Dag1 knockouts with GFAP-Cre, Mox2-Cre, and nextin Cre, crossing the Dag1 floxed mice with a Emx1-Cre line [48] effectively abrogated glycol-α-DG, indicated by loss of IIH6 antibody signals (Figure S7), and resulted in disturbed cortical neuronal organization (Figure 7A). While the neuronal dysplasia was more frequently observed medially in the cingulate cortex, neuronal lamination in the neocortex of the Dag Emx1-Cre+ cKO mice was largely preserved: Most of the earlier born neurons marked by the Foxp2 antibody were observed in the deeper cortex as expected; a majority (∼80%) of Cux1+ later born neurons was able to migrate to superficial layer II/III (Figure 7B–D). In contrast to the grossly laminated Nde1^−/−^ and Dag Emx1-Cre+ cKO cortices, neurons in the neocortex of the Nde1^−/−^; Dag Emx1-Cre+ cKO double mutant mice showed little discernable lamina organization (Figure 7A): Widespread neuronal dysplasia were observed in the deep cortex as well as on the cortical margin with cobblestone-like focal ectopic neurons occupying the MZ (Figure 7A,B). Further characterization of neuronal lamination defects with cortical layer-specific markers Cux1 and Foxp2 showed that the neocortex of the Nde1^−/−^; Dag1 cKO double mutant was not only disorganized but also partially inverted. Over 50% of the earlier born Foxp2+ deep layer neurons were mislocalized to the outer half, whereas the later born Cux1+ superficial layer neurons showed a completely un-laminated distribution (Figure 7B–D). Though these neuronal migration defects indicated failures of later born neurons to migrate past their earlier-born predecessors, a small number of Foxp2+ or Cux1+ neurons were found to have over-migrated into the MZ, where they further differentiated and became recognizable by the NeuN antibody (Figure 7E). The synthetic phenotype of Nde1 and Dag1 mutations indicated that the Lis1-Nde1 and DGC complexes function synergistically in the RGC during cortical development. Because Nde1 directly interacts Dmd/Utrn to which Dag1 also binds, the mechanism underlying this synergistic function should be a collaborative stabilization of a multi-protein complex required for maintaining the cell surface integrity. The fact that the double mutant showed both enhanced under- and over-migration of cortical neurons also supports the notion that a Lis1-Nde1-DGC multi-protein complex regulates neuronal migration non-cell-autonomously in the RGC rather than in migrating neurons, and that the key cell developmental defect underlying the disorganized cortical layering in lissencephaly is a non-cell-autonomous malfunction of the RGC scaffold.

### Unremarkable Impairment of Cell-Autonomous Housekeeping Functions

Although LIS1, Nde1, as well as its related Ndel1 have been implicated in cell-autonomous housekeeping functions, such as modulating the dynein motor complexes through in vitro analyses, Nde1 appears to be preferentially required by the CNS and the loss of functional phenotypes of Lis1-Nde1 that we observed are highly tissue-, developmental-stage-, and cell-type-specific. Despite the aberrant basal-lateral morphology and adhesion, the mutant RGCs preserved the normal radial glial polarity and apical-basal compartment. Besides unaltered adherence junctions (AJs) and basal bodies marked by β-catenin and pericentrin, respectively [18], distributions of apical protein Pals1 and basal-lateral membrane protein Na-K ATPase in the Lis1^+/−^ Nde1^−/−^ RGCs were indistinguishable from those of control RGCs (Figure S8A,B). In contrast to their severely impaired functions in cortical development, Lis1^+/−^ Nde1^−/−^ mutant progenitors isolated from the mutant cortex showed remarkably improved behaviors in culture. Although these progenitors grew slower and often formed smaller neurospheres, they did not show increased spontaneous differentiation in culture. Upon growth factor withdraw, they differentiated into astroglial cells with normal morphology as well as neurons with long elaborated processes and fine growth cones (Figure S8C). Because cells derived from Lis1^+/−^ Nde1^−/−^ progenitors in culture were completely indistinctive to those from the wild type progenitors, it is suggested that Lis1-Nde1 deficiency did not impair the basic cytoskeleton and motor functions. Although the Lis1^+/−^ Nde1^−/−^ mutants die of feeding difficulties shortly after birth with severely malformed CNS and atrophic muscles, they were born at the Mendelian ratio with unremarkable changes in the size and structure of most of the organs [18]. These together indicated that fundamental cellular functions, such as the proliferation and differentiation of cells outside of the CNS, along with intracellular organelle positioning and axonal protein transport of cells in the CNS, were largely undamaged by the Lis1^+/−^ Nde1^−/−^ mutation. Therefore, the key mechanism by which the Lis1-Nde1 complex controls CNS development is cell type and tissue context dependent.

The precocious neurogenesis in Lis1^+/−^ Nde1^−/−^ RGCs led to a large number of ectopic Cajar-Retzius cells and dramatically increased ECM glycoprotein Reelin secreted by Cajar-Retzius cells. Nonetheless, the pial BM breach and neuronal over-migration was not a result of elevated Reelin, as abrogating Reelin by Reln heterozygous and homozygous mutations did not rescue the cobblestone lissencephaly–like phenotype (Figure S9).

## Discussion

**Figure 8:**
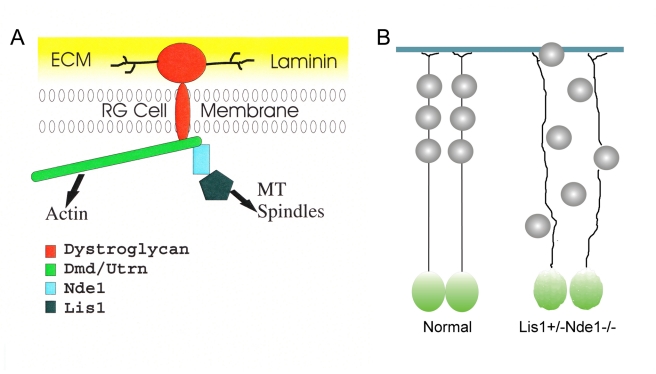
The 3-D regulation of RGC function by the Lis1-Nde1-DGC complex. (A) Molecular organization of the Lis1-Nde1-DGC complex in RGC. Limited members of Lis1-Nde1-DGC complex are depicted. Additional molecules that may also be associated with this complex are omitted. (B) A schematic presentation of RGC defects and developmental cortical malformations caused by Lis1-Nde1 deficiency.

In summary, we presented compelling evidences for a Nde1-depedent mechanism that specifically stabilizes the DGC in RGCs. The formation of the Lis1-Nde1-DGC multi-protein complex allows the establishment of a physical link between the Lis1-Nde1 regulated mitotic apparatus and the DGC associated cell surface to control the mitotic cell shape, spindle orientation, as well as the proper cytoarchitecture and neurogenic niche of RGCs. Impaired function of Lis1-Nde1-DGC leads to dramatically increased asymmetric divisions, leading to the reduction of progenitor pool. Meanwhile, the complex is essential for maintaining the lateral adhesion of basal processes of the RGCs, which serve as the infrastructure for neuronal migration; loss of such function results in the coexistence of both “under”- and “over”-migration of cortical neurons (Figure 8). Therefore, a three-dimensional regulation of the morphology, cell-cell adhesion, and cytoarchitectures of the RGCs determines their neurogenic fate and the destination of their daughter neurons, which in turn determine the size and shape of the cerebral cortex. This study provides direct evidence of a non-cell-autonomous regulation of cortical neuronal migration by RGCs and also for the first time, to our knowledge, shows how one protein complex is able to integrate two different but tightly coupled essential functions of the RGC, providing a mechanistic basis for the co-existence of neurogenesis and neuronal migration defects in lissencephaly syndromes.

### Multifaceted Role of Nde1 in Regulating RGC Functions

Identified as the essential physical and CNS functional partner of LIS1, Nde1 is a cytoplasmic scaffold whose subcellular localizations may be dynamically regulated by the cell cycle and cell's activity or physiological conditions [15]. By interacting with centrosomal and microtubule associated proteins, Nde1 plays roles in the organization of microtubules and the assembly of the mitotic spindle [14],[15]. Nde1 null mutation resulted in aberrant mitotic spindle function/orientation specifically in RGCs, which led to increased asymmetrical division in mid-corticogenesis [15]. Regardless of the role of Nde1 as a centrosomal scaffold, centrosomes, basal bodies, as well as Nde1's centrosomal partner, Pericentrin, remained intact in Lis1-Nde1 double mutants [18], suggesting a non-housekeeping regulatory requirement of Nde1 in cortical development. In this study, we illustrated a new functional site of Nde1 at the basal-lateral surface of RGCs, where Nde1 interacts simultaneously with Lis1 and Dmd. The cell-surface-bound Nde1 not only provides a stable anchorage for astral microtubules to the cell cortex to determine mitotic spindle orientations during RGC mitosis, but also regulates the function of RGCs and in a number additional ways (1) establishes cell-cell or cell-ECM contacts to stabilize the microenvironment of RGCs and allows them to sense the proper cell surface signals; (2) maintains the plasma membrane integrity to permit RGCs extending radially into extremely long cells during the course of cortical neurogenesis and neuronal migration; and (3) ensures the adhesion of newborn cortical neurons on the basal processes of the RGC and guides their migration. Therefore, the ability of Nde1 in cross-linking the ECM, the plasma membrane, the cortical actin cytoskeleton, and the mitotic spindle is essential for its role in determining the mitotic mode, the mechanical strength, the differentiation state of RGCs, as well as its service as supplier and transporter of cortical neurons. Our findings are perfectly in line with the recent identification of *NDE1* as one of the most essential genes that govern the developmental formation of the cerebral cortex.

Although the current study demonstrates clearly that Lis1-Nde1 cooperates with the DGC at the basal lateral surface of RGC to integrate cortical neurogenesis and neuronal migration, loss of Nde1 is not equivalent to Dmd or dystroclycan deficiency in both mice and men. The differential phenotype between Nde1 and DGC as well as the dynamic features of Nde1 protein also suggest DGC independent mechanisms and functions of Nde1. Nde1 is a mitotic phospho-protein and a functional substrate of Cdk1 during cell division [16]; its subcellular localization is cell cycle dependent and may be altered by phosphorylation. Thus, it is well conceivable that there are other subcellular sites of Nde1 action in addition to the cell surface. Therefore, further exploring both DGC-dependent and DGC-independent functions of Nde1 will be essential for understanding how Nde1 safeguards the RGC during cerebral cortical development.

### Shared Requirement of DGC in RGCs and Muscles

The identification of Nde1-Utrn/Dmd interaction also provided insight into the molecular function of dystrophin. Aside from being an essential molecule at the membrane-cytoskeleton interface of muscle cells, one-third of DMD patients also exhibit significant developmental cognitive and behavioral abnormalities including infantile autism, attention deficit spectrum disorders, and mental retardation [49],[50], suggesting that dystrophin also plays an important role in brain development. As a gene with 79 exons spanning 3.4 Mb, *DMD* is under complex transcriptional regulation with the presence of many internal promoters and isofroms that are expressed in various tissue and developmental-dependent patterns [32],[51]. At least two shorter non-muscle products, Dp140 and Dp71, which share the common cystein-rich and carboxy-terminus domains with the full-length dystrophin, have been detected in the brain [50],[52]. A deficit in Dp140, expressed mainly in fetal brain tissues, is strongly associated with the neuropsychological abnormalities of DMD [52]. Our study now demonstrates that Nde1 interacts with the C-terminus cystein-rich domain of all dystrophin isoforms and that dystrophin and Dp140 are part of the Lis1-Nde1-based multiprotein complex essential for RGC functions in cortical neurogenesis and neuronal migration. Although muscle cells and RGCs are very different types of cells and perform distinctive functions, they share a unique set of structural and functional features. Both muscle fibers and RGCs are cells with long slender morphology, both engage in strong cell-cell interactions with their nearest neighbors, both undergo constant dynamic active movements of contraction or division that require high fidelity maintenance of their plasma membranes, and both present nuclei migration activities either in a cell cycle dependent or in a cell fusion and differentiation dependent fashion. These common characters and activities raise the possibility of common cell biology mechanisms regulated by the Lis1-Nde1-DGC complex in muscle fibers and RGCs. Our data are well in line with the general understanding of the function of dystrophin as a cytolinker that binds multiple components of the filamentous cytoskeleton to protect the sarcolemma from mechanically induced damage. Our data are also in parallel to the recent finding that dystrophin may directly interact with costameric microtubules and regulate microtubule integrity and organization [53].

### Common RGC Defects in Type I and Type II Lissencephaly

Lissencephaly caused by LIS1 haploinsufficiency has been largely attributed to cell-autonomous neuronal motility defects due to the association of LIS1 with microtubules and microtubule associated motors [54],[55], while type II lissencephaly has so far been thought to be caused primarily by instability of the cortical pial BM that subsequently leads to overmigration of cortical neurons into the subarachnoid space [42],[56]. Nevertheless, both classes of lissencephaly syndromes are primarily malformations of the CNS that may involve muscle anomalies, and both share the general brain pathology of a smooth cerebral surface, reduced brain size, and severely disrupted cortical neuronal lamination. The extreme microcephaly, contrasted by many well-formed organs outside of the CNS and the co-existence of severe migration arrest and over-migration of cortical neurons in the Lis1-Nde1 double deficient mice, suggests that the neuronal migration defects associated with lissencephaly do not reflect a motility incompetence of the mutant neurons, but rather a non-cell-autonomous guidance error from the RGCs. The notion of neural migration defects caused by the misguidance from RGCs was also supported by experimental observations of aberrant radial glial fibers in the Lis1 mutant mice [57]. Moreover, data in this study suggest that the major molecular defect underlying the aberrant migration guidance is the loss of plasma membrane mechanical strength and adhesion of RGC's long basal-lateral surface due to destabilized Lis1-Nde1-DGC complexes. Thus, the common pathogenic mechanism underlying lissencephaly is impaired RGC functions.

Although ECM molecules in the cortical pial BM are thought to be produced by the meningeal fibroblasts, RGCs contribute significantly to the integrity of BM structure. The BM in the Lis1-Nde1 mutant mice was well assembled before the onset of neurogenesis, and only deteriorated after E13.5, when RGCs failed to elongate and provide adequate support to both the migrating neurons and the BM. Therefore, molecular complexes between Lis1-Nde1 and DGC on the basal lateral membrane of RGCs are compositely required for establishing proper neuron-radial glial and radial glial-BM interfaces to promote the radial extension of RGCs, to maintain the stability of pial BM, and to control the precise final location of cortical neurons.

The concept of BM maintenance by RGCs is supported by data from analyzing mouse mutations of FAK, IlK, and Gpr56. While deletion of FAK in newborn cortical neurons was insufficient to induce migration defects, targeting meningeal fibroblasts led to the formation of aberrantly positioned neurons non-cell-autonomously. A more striking type II lissencephaly-like neuronal lamination phenotype only resulted from targeted deletion of FAK from RGCs [58]. Similarly, conditional knocking out of Integrin-linked kinase (Ilk) in RGC but not in neurons resulted in fragmentation of BM and disturbance of neuronal lamination [59]. Ilk is a kinase apparently required for modulating cell adhesion by linking integrin to the actin cytoskeleton. Ablation of Ilk in RGCs led to severely malformed radial glial basal fibers and retracted radial glial endfeet that are reminiscent of Lis1-Nde1 double deficient RGCs, suggesting common functions in regulating RGC surface integrity and supporting pial ECM assembly and stability. More recently, mouse mutations of Gpr56, a gene in which mutations underlie the regional frontal-parietal lobes malformation of the human cerebral cortex BFPP, also showed type II lissencephaly-like phenotypes [60]. Although the pathology of *GPR56* mutation in humans is unclear, Gpr56 is selectively expressed in the RGC [61]. As a newly identified orphan G protein-coupled receptor, GPR56 contains a long N-terminal ecto-domain that may putatively mediate cell-cell and cell ECM adhesion of RGCs. Thus, aberrant RGC morphology and adhesion may underlie multiple forms of cerebral cortical malformation disorders including both type I and type II lissencephaly syndromes and bilateral frontoparietal polymicrogyria (BLPP). The differences in disease manifestation may be largely due to the differential requirements of specific gene complexes in different membrane submicrodomains along the long radial processes of RGCs or to the spatial-temporal gradients of cell adhesion molecules and their ligands.

### Specific Features of RGC and Cerebral Cortical Morphogenesis

The cerebral cortex is an evolutionarily recent structure characterized by extraordinarily high neuronal density and organization. Neurogenesis and neuronal migration are precisely coordinated to allow for the efficient, ordered generation and transportation of neurons to designated cortical layers within a relatively narrow window of embryonic development. During the course of mammalian evolution, the size of the cortex and the number of cortical neurons increase exponentially. The increased neuronal production has been granted to the increased number and symmetrical divisions of the neural progenitor cells, however little is known about mechanisms specifically required for higher mammals to expand their progenitor pool with higher efficiency and fidelity.

The unique feature that differentiates cortical neural progenitors from neuronal progenitors from other parts of the nervous system or lower vertebrates is their extraordinary elongated radial glial morphology. Although vast experimental studies have demonstrated that RGCs utilize a set of conserved mechanisms belonging to lower vertebrates to regulate their rate and mode of cell divisions, such as controlling the orientation of mitotic spindles and the inheritance of polarized cell fate determinants or mother-daughter centrosomes, none of the previously described mechanisms has taken the distinctive morphology and cytoarchitectural features of RGC into consideration. Mounting evidence has suggested that the size of the cerebral cortex is determined by the timing of NEC transformation into RGC [62]–[64]. Longer RGCs are the evolutionary by-product of a larger cerebral cortex. Therefore, understanding molecular machineries that are specifically required for RGC formation, maintenance, radial extension, and function may provide answers on how the cerebral cortex has evolved. Although analyses presented in this article are somewhat limited by a mouse model of cerebral cortical malformation diseases, this genetic model largely recapitulated the pathology of human patients with impaired LIS1 and DGC, as well as homozygous *NDE1* loss of functions [16],[17]. By identifying a molecular complex that integrates cerebral cortical neurogenesis with neuronal migration, this study, to our knowledge, is the first demonstration on how CNS-specific regulation may be achieved by genes that regulate the distinctive cell biological features of the building blocks of the CNS. One interesting note is the fact that the Lis1-Nde1-DGC complex appears to be specifically essential for the long fiber-like RGCs and muscle cells. This suggests that the molecular pathways centered by the Lis1-Nde1-DGC complex are more essential for the neurogenesis and morphogenesis of primates and humans as their RGCs are much longer. This may be why loss of *NDE1* functions has a much stronger impact in humans than in mice. Therefore, further exploring cell molecular mechanisms that are specifically required by NDE1 to coordinate the structure and function of RGCs will give an opportunity to understand how the cerebral cortex expands throughout evolution.

## Materials and Methods

### Mouse Genetics

Lis1^+/−^ Nde1^−/−^ and Lis1^+/−^ Nde1^+/−^ mice were obtained by standard genetic crosses of Lis1^+/−^ and Nde1^+/−^ mice as described [18],[65]. The Exm1-Cre and Dag1 cKO(floxed) mice were obtained from JaxMice. Nde1^−/−^ and Dag1cKO Emx1-Cre double mutants were generated by standard genetic crosses. All mice were housed and bred according to the guidelines approved by the ACUC committee at Northwestern University. For timed matings, the day of the vaginal plug was considered E 0.5.

### Immunohistology

Immunohistology was performed as described [18] with 12 µm frozen sections. For detecting cell surface associated Nde1 in tissue sections, fresh mouse embryonic brains were embedded in OCT. Frozen sections were prepared at 14 µm, fixed in acetone for 2 min, air dried for 20 min at room temperature, and then immunostained in PBS plus 0.25% Saponin. Antibodies used are as follows: Dystrophin (Santa Cruz, Developmental Study Hybridoma Bank, Abcam), β-DG (Vector Lab), α-DG (IIH6C4, VIA4-1, Millipore), GLAST, BLBP, Calretinin (Millipore), MPM-2 (Upstate), β-catenin (Transduction Lab), Utrn (Vector Lab, Developmental Study Hybridoma Bank), RC2, Nestin, Na-K ATPase (Developmental Study Hybridoma Bank), Laminin (Millipore), Nidogen (Calbiochem), Tuj-1 (Abcam), DCX [15], Map2, α-Tubulin (Sigma), GFAP (DAKO), Dynein IC (Millipore), Pals 1 (Epitomics), Cux1 (Santa Cruz), Foxp2 (Abcam), monoclonal mouse anti-Anti-Flag, anti-myc, and rabbit anti-Myc (GenScript), and monoclonal anti-EGFP(Clontech).

### Ultra-Structural Analysis

Fresh mouse embryos at E12.5 and E14.5 were fixed in 2% glutaraldehyde and processed for standard transmission electron microscopy analysis. Specimens were examined with a JEOL 1220 transmission electron microscope equipped with Kodak digital camera.

### Cortical Lysates and Immunoblotting

Cerebral cortices were dissected from mouse embryos and flash frozen in liquid nitrogen. Upon obtaining genotype information, cortical samples were homogenized in 95°C SDS sample buffer. Approximately 25 µg total protein from each sample was used for immunoblotting analyses. The loading was adjusted by using Tubulin or β-catenin as controls. Quantitative analysis of immunoblots was performed with Image J.

### Yeast Two Hybrid Screen

Yeast two hybrid screen was performed as described [14].

### Plasmids

Various Nde1, Utrn, and Dmd fragments were generated by PCR amplification of the mouse full-length Nde1, Utrn [66], or Dmd. Each PCR product was first cloned into PCRII (Invitrogen), sequenced, and then subcloned to pcDND3.0 (Invitrogen) for mammalian expression, to pEGFP (Clontech) for N-terminal EGFP fusion and mammalian expression, or to pGEX2T (Pharmacia) for GST fusion and bacterial expression. Flag-Dmd pcDNA3 was generated from pBastBac1-Dmd [67] by subcloning the NotI-SmaI fragment of full-length Dmd cDNA into pcDNA3.0 with modified poly-cloning sites between HindIII and ApaI.

### Cell Culture, Immunostaining, and Immunoprecipitation and GST Pull-Down

Hela and 293T cells were cultured in DMEM with 10% FBS. SCC9 cells were cultured in DMEM/F12 with 10% FBS. All immunofluorescence cell stainings were performed by fixation with 4% EM grade formaldehyde(Ted Pella), and permeabilization with 0.25% Saponin in a staining solution containing 25 mM HEPES, pH 7.4; 2.5 mM MgAc2, 25 mM KCl, and 250 mM Sucrose. For immunoprecipitation analyses, 293T cells were co-transfected with plasmids encoding Flag-Utrn [66], Flag-Dmd, myc- or EGFP-tagged Lis1, Nde1, or Utrn, Dmd, and Nde1 truncation constructs. Cells were lysed in 150 mM NaCl, 50 mM Tris, pH 7.5, 0.1% TX100, 1 mM DTT, 20 u/ml DNAase I; 25 µg/ml pepstatin A, 25 µg/ml leupeptin, 25 µg/ml Aprotinin, 10 mM Benzamidine, and 2 mM PMSF. Immunoprecipitations were performed with monoclonal anti-myc (9E10) or anti-flag antibodies and protein A/G sepharose. The immunocomplexes were washed 4–6 times with the lysis buffer and analyzed by immunoblotting with anti-Flag, anti-EGFP, or rabbit anti-myc antibodies. GST pull-down was performed as described [14].

## Supporting Information

Figure S1 Recombinant Utrn can be expressed at a higher level by Nde1 co-transfection. Western blotting analysis showing that the level of recombinant Utrn expressed in Cos7 cells was higher when it was co-transfected with Nde1.
(TIF)

Figure S2 Quantitative representation of β-DG protein levels detected by immunoblotting. Data were collected from total protein extracts from 3 litters of Lis1, Nde1 mutant embryos at E12.5.
(TIF)

Figure S3 Subtle alteration of glycol-α-DG before the onset of cortical neurogenesis. Double immunohistological staining of E10.5 mouse embryos with antibodies to glycol-α-DG (in red) and Nestin (in green). The level and distribution of glycol-α-DG between Nde1^+/−^Lis1^+/+^ and Nde1^−/−^Lis1^+/−^ cortices were almost indistinguishable.
(TIF)

Figure S4 Double immunohistological staining of E12.5 mouse embryos with antibodies to glycol-α-DG (in red) and β-Catenin (in green), showing that reduced glycol-α-DG (in red) in the Nde1^−/−^Lis1^+/−^ neocortical VZ was first detected at E12.5, shortly after the onset of cortical neurogenesis.
(TIF)

Figure S5 Substantial amount of programmed cell death, identified by cleaved caspase 3 immunostaining (green), was detected in the neocortex of Lis1^+/−^ Nde1^−/−^ mutant at E11.5, but largely disappeared after E14.5.
(TIF)

Figure S6 Muscle developmental defects caused by the Nde1^−/−^Lis1^+/−^ mutation. H&E stained transverse and longitudinal sections of muscles in the hind limb of the Nde1^−/−^Lis1^+/−^ mutant and their control littermates at birth. 5 Nde1^−/−^Lis1^+/−^ mutants and 3 littermate control samples were analyzed; representative figures were shown. Muscle atrophy and fibrosis were typically observed in the Nde1^−/−^Lis1^+/−^ mutant, suggesting muscular dystrophy-like pathology.
(TIF)

Figure S7 Effective abrogation of DG by the Emx1-Cre. Glyco-α-DG could be abrogated effectively in the developing cerebral cortex by crossing the Dag1 floxed mice with the Emx1-Cre line. Spatially matched brain sections of E12.5 embryos were immunostained by the anti-αDG IIH6 monoclonal antibody (red). Conditional knocking out of Dag1 by the Emx-1 Cre resulted in absence of IIH6 immunosignals in the cerebral cortex. This result also demonstrated that the IIH6 immunohistological signals presented in this study were highly specific.
(TIF)

Figure S8 Unremarkable changes in vital cellular functions by Lis1^+/−^Nde1^−/−^ mutation. (A, B) Immunoblotting and immunohistological analysis of E13.5 cortical protein and brain sections showed that the level and distribution of apical protein Pals 1 (green) and basal-lateral membrane protein Na-K ATPase were unaltered by the Lis1^+/−^Nde1^−/−^ mutation, which suggested that Lis1^+/−^Nde1^−/−^ RGCs retained the correct apical-basal polarity and membrane compartments of normal RGCs. (C) Lis1^+/−^Nde1^−/−^ mutant progenitors were isolated from the cerebral cortex of E12.5 embryos and cultured as neurospheres in DMEM/F12 supplemented with N2, 10 nM bFGF, and 20 nM EGF for 2 wk to 6 mo. After growth factor withdraw, neurons and astalglial cells derived from Lis1^+/−^Nde1^−/−^ progenitors showed little structural difference from wild type cells in culture.
(TIF)

Figure S9 The Reelin independence of BM fragmentations in the Lis1^+/−^Nde1^−/−^ mutants. Brains of Lis1^+/−^Nde1^−/−^Reln^+/+^, Lis1^+/−^Nde1^−/−^Reln^+/−^, and Lis1^+/−^Nde1^−/−^Reln^−/−^ mice were analyzed immunohistologically with antibodies to laminin to highlight the BM (in red) and Calretinin to label C-R cells (in green). BM fragmentations and C-R cell ectopia were observed in all three mutants despite the fact that they expressed different levels of Reelin. Thus, the RGC basal-lateral morphology defect caused by in the Nde1^−/−^Lis1^+/−^ mutation was not due to the elevated Reelin in the mutant cortex.
(TIF)

## Abbreviations

BM: basement membrane
cKO: conditional knockout
CNS: central nervous system
CP: cortical plate
DG: dystroglycan
DGC: dystrophin/dystroglycan glycoprotein complex
ECM: extracellular matrix
MZ: marginal zone
NEC: neural epithelial cell
RGC: radial glial cell
SVZ: subventricular zone
VZ: ventricular zone
