# Supplementary figures and images for: Three-Dimensional Regulation of Radial Glial Functions by Lis1-Nde1 and Dystrophin Glycoprotein Complexes
Source: PLoS Biol. 2011 Oct 18;9(10):e1001172. doi: 10.1371/journal.pbio.1001172 (PMC3196477; doi:10.1371/journal.pbio.1001172)

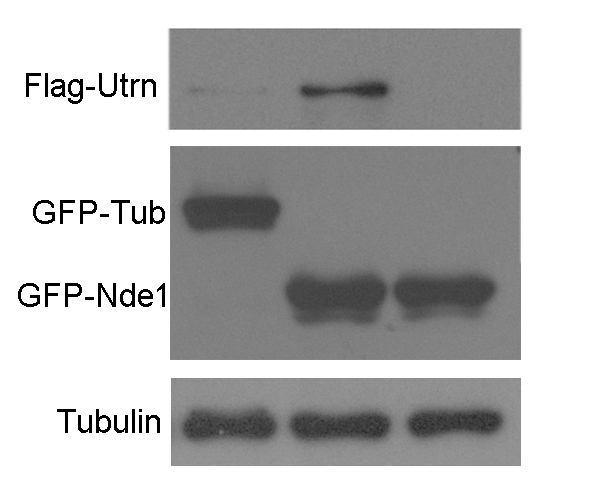

Supplement: Figure S1 — Recombinant Utrn can be expressed at a higher level by Nde1 co-transfection. Western blotting analysis showing that the level of recombinant Utrn expressed in Cos7 cells was higher when it was co-transfected with Nde1. (TIF) [file pbio.1001172.s001.tif]

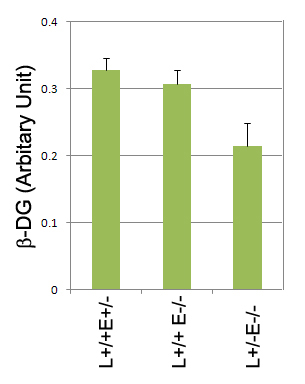

Supplement: Figure S2 — Quantitative representation of β-DG protein levels detected by immunoblotting. Data were collected from total protein extracts from 3 litters of Lis1, Nde1 mutant embryos at E12.5. (TIF) [file pbio.1001172.s002.tif]

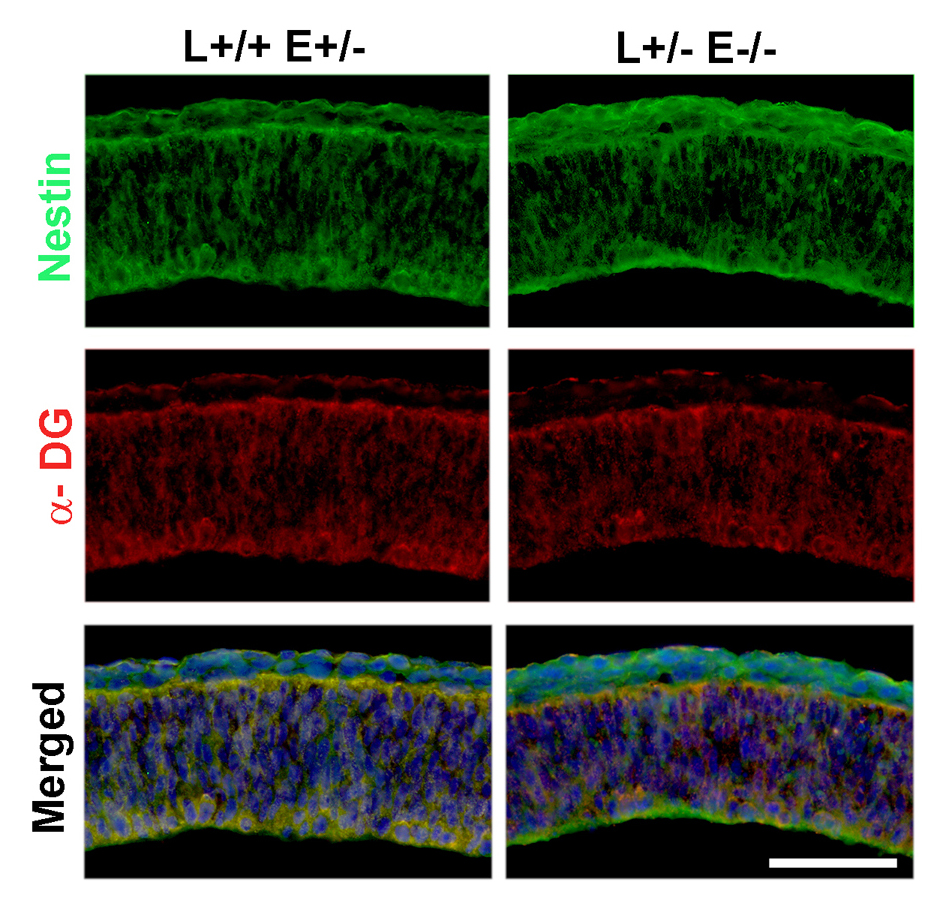

Supplement: Figure S3 — Subtle alteration of glycol-α-DG before the onset of cortical neurogenesis. Double immunohistological staining of E10.5 mouse embryos with antibodies to glycol-α-DG (in red) and Nestin (in green). The level and distribution of glycol-α-DG between Nde1+/−Lis1+/+ and Nde1−/−Lis1+/− cortices were almost indistinguishable. (TIF) [file pbio.1001172.s003.tif]

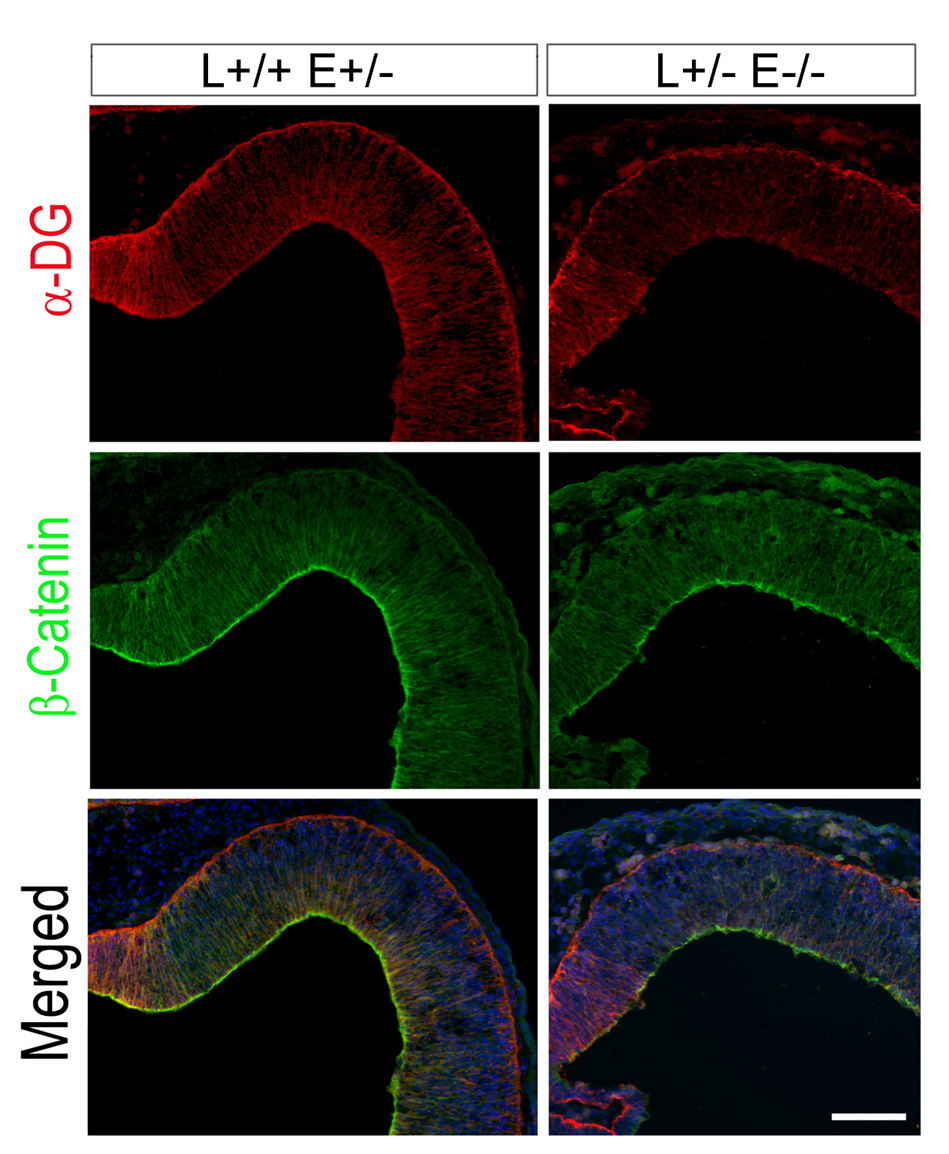

Supplement: Figure S4 — Double immunohistological staining of E12.5 mouse embryos with antibodies to glycol-α-DG (in red) and β-Catenin (in green), showing that reduced glycol-α-DG (in red) in the Nde1−/−Lis1+/− neocortical VZ was first detected at E12.5, shortly after the onset of cortical neurogenesis. (TIF) [file pbio.1001172.s004.tif]

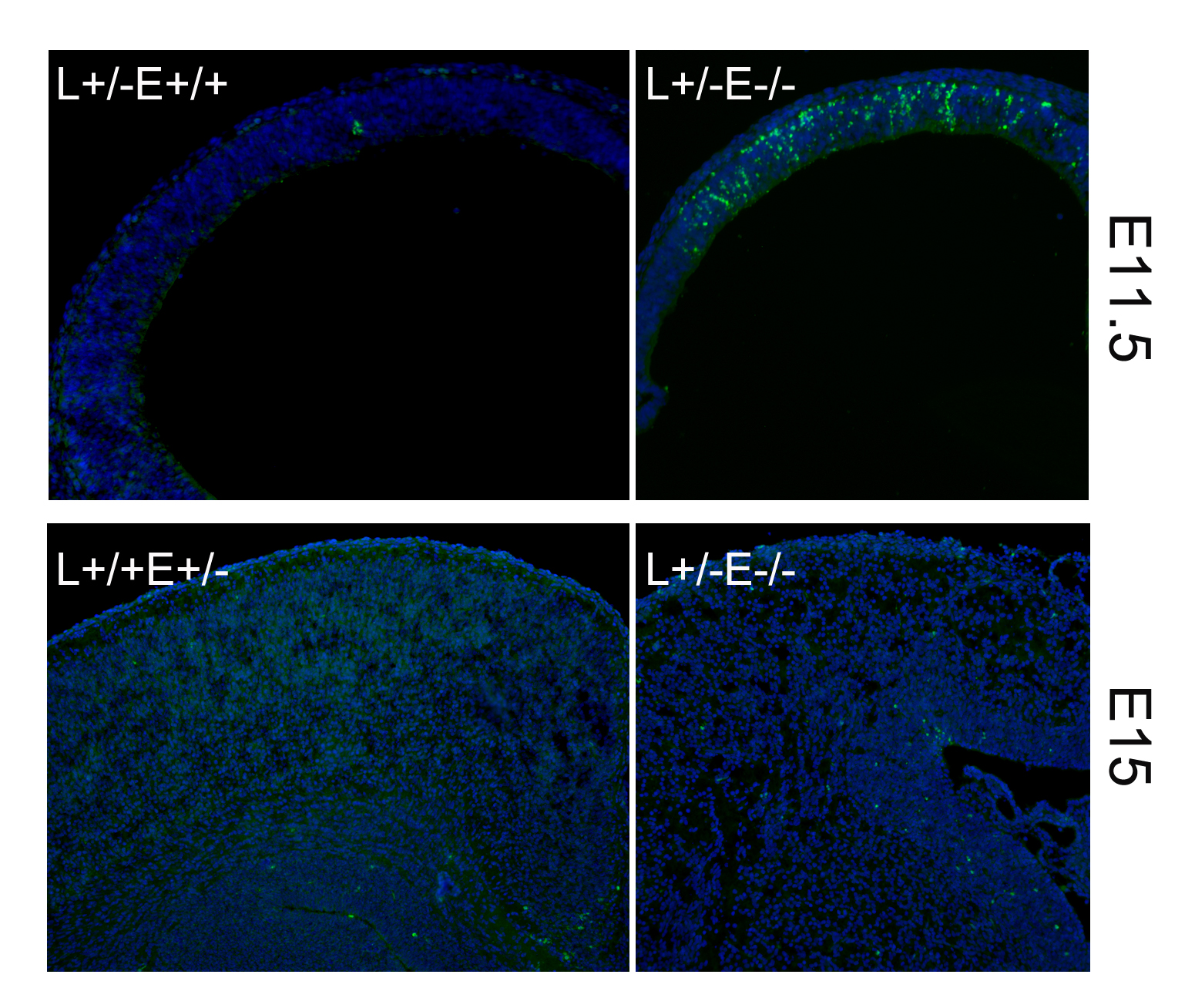

Supplement: Figure S5 — Substantial amount of programmed cell death, identified by cleaved caspase 3 immunostaining (green), was detected in the neocortex of Lis1+/− Nde1−/− mutant at E11.5, but largely disappeared after E14.5. (TIF) [file pbio.1001172.s005.tif]

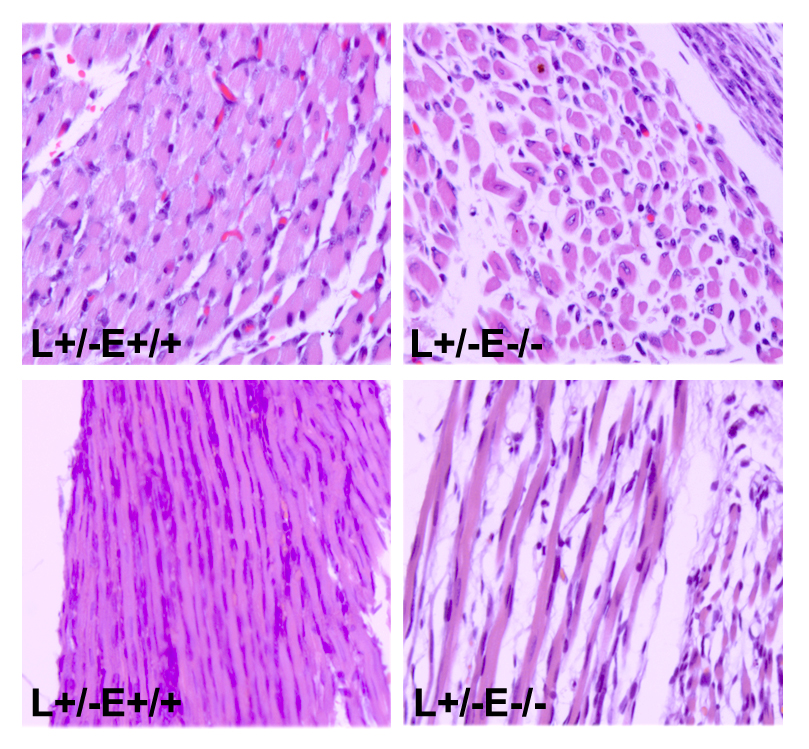

Supplement: Figure S6 — Muscle developmental defects caused by the Nde1−/−Lis1+/− mutation. H&E stained transverse and longitudinal sections of muscles in the hind limb of the Nde1−/−Lis1+/− mutant and their control littermates at birth. 5 Nde1−/−Lis1+/− mutants and 3 littermate control samples were analyzed; representative figures were shown. Muscle atrophy and fibrosis were typically observed in the Nde1−/−Lis1+/− mutant, suggesting muscular dystrophy-like pathology. (TIF) [file pbio.1001172.s006.tif]

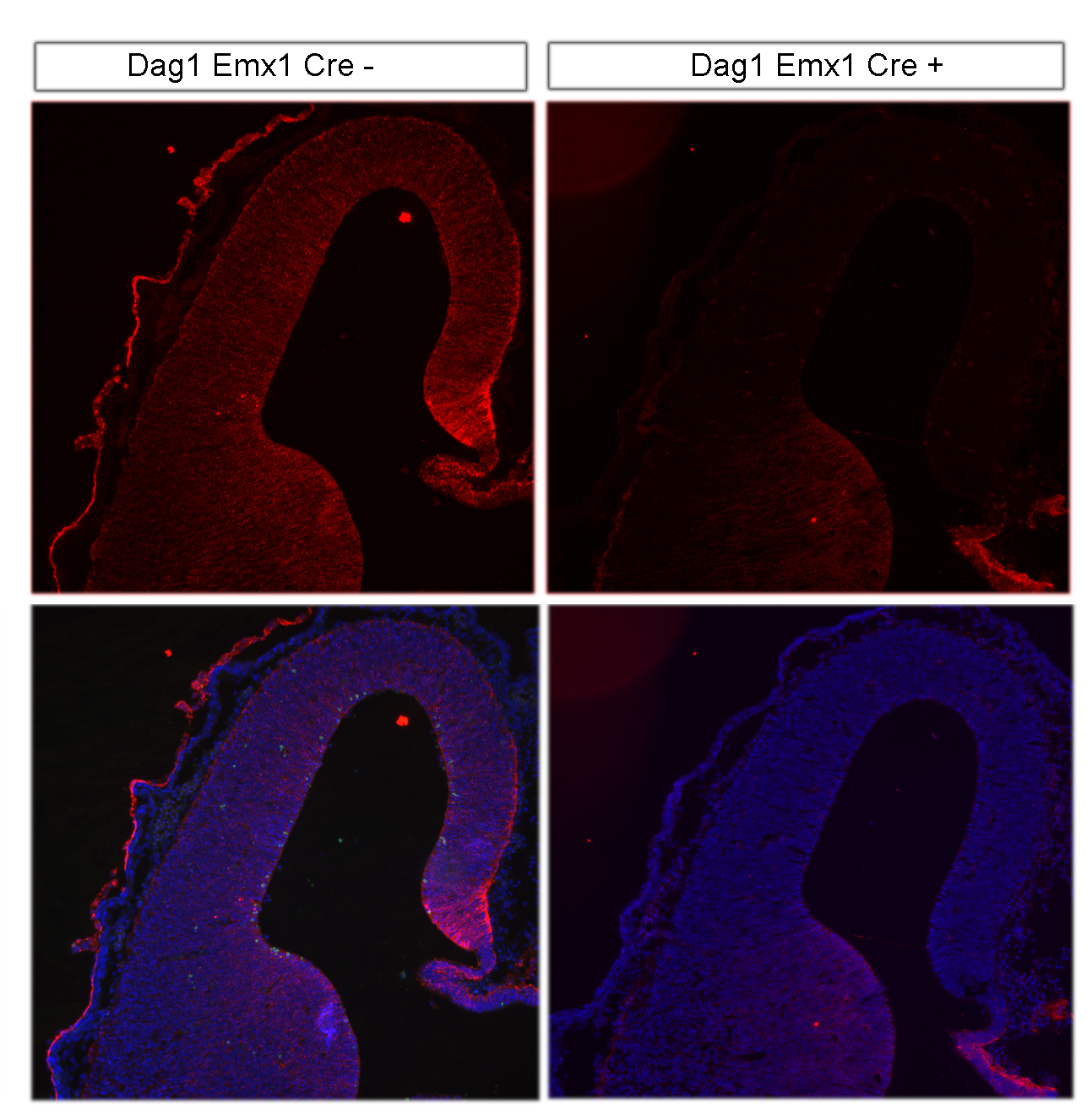

Supplement: Figure S7 — Effective abrogation of DG by the Emx1-Cre. Glyco-α-DG could be abrogated effectively in the developing cerebral cortex by crossing the Dag1 floxed mice with the Emx1-Cre line. Spatially matched brain sections of E12.5 embryos were immunostained by the anti-αDG IIH6 monoclonal antibody (red). Conditional knocking out of Dag1 by the Emx-1 Cre resulted in absence of IIH6 immunosignals in the cerebral cortex. This result also demonstrated that the IIH6 immunohistological signals presented in this study were highly specific. (TIF) [file pbio.1001172.s007.tif]

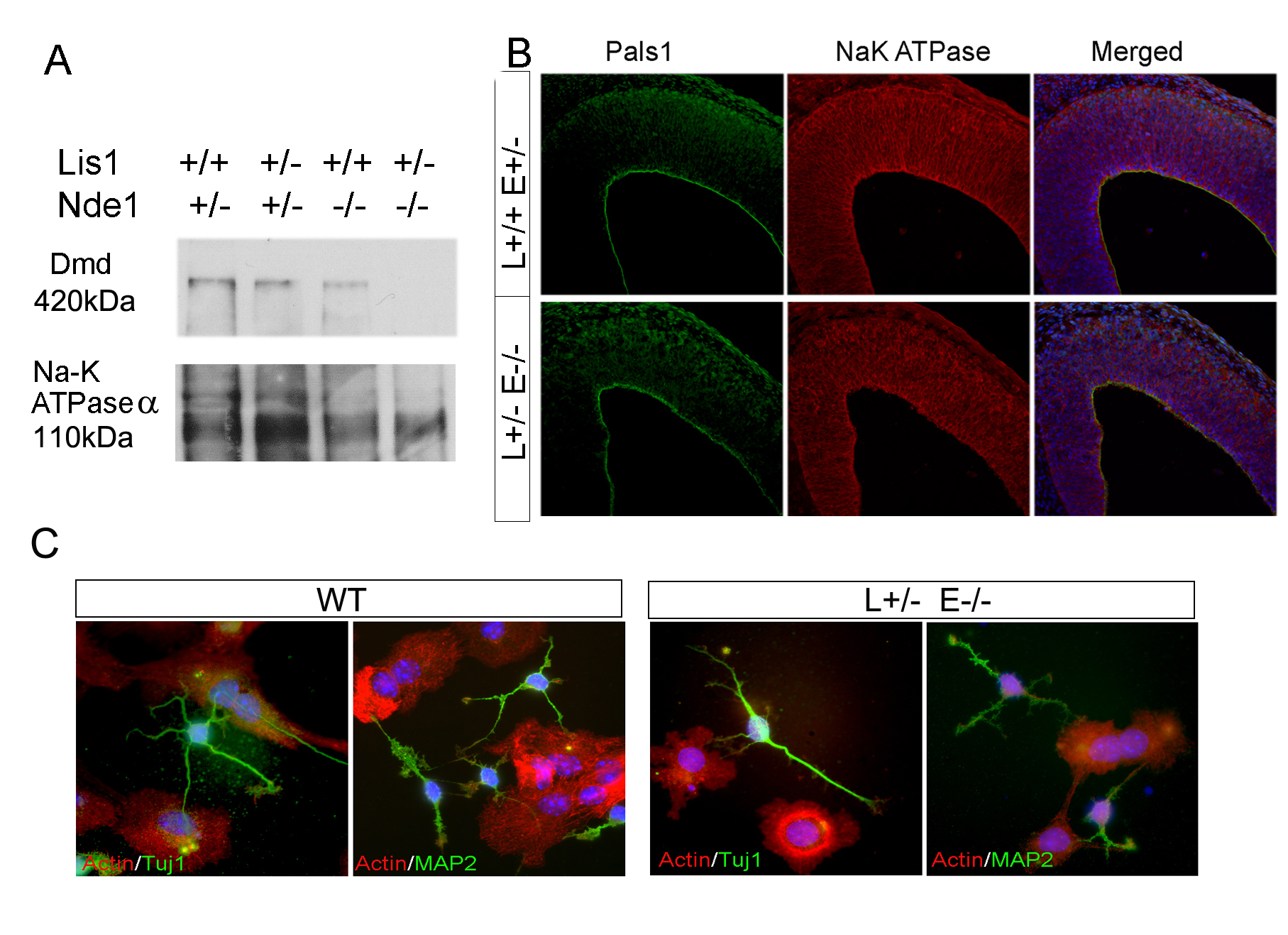

Supplement: Figure S8 — Unremarkable changes in vital cellular functions by Lis1+/−Nde1−/− mutation. (A, B) Immunoblotting and immunohistological analysis of E13.5 cortical protein and brain sections showed that the level and distribution of apical protein Pals 1 (green) and basal-lateral membrane protein Na-K ATPase were unaltered by the Lis1+/−Nde1−/− mutation, which suggested that Lis1+/−Nde1−/− RGCs retained the correct apical-basal polarity and membrane compartments of normal RGCs. (C) Lis1+/−Nde1−/− mutant progenitors were isolated from the cerebral cortex of E12.5 embryos and cultured as neurospheres in DMEM/F12 supplemented with N2, 10 nM bFGF, and 20 nM EGF for 2 wk to 6 mo. After growth factor withdraw, neurons and astalglial cells derived from Lis1+/−Nde1−/− progenitors showed little structural difference from wild type cells in culture. (TIF) [file pbio.1001172.s008.tif]

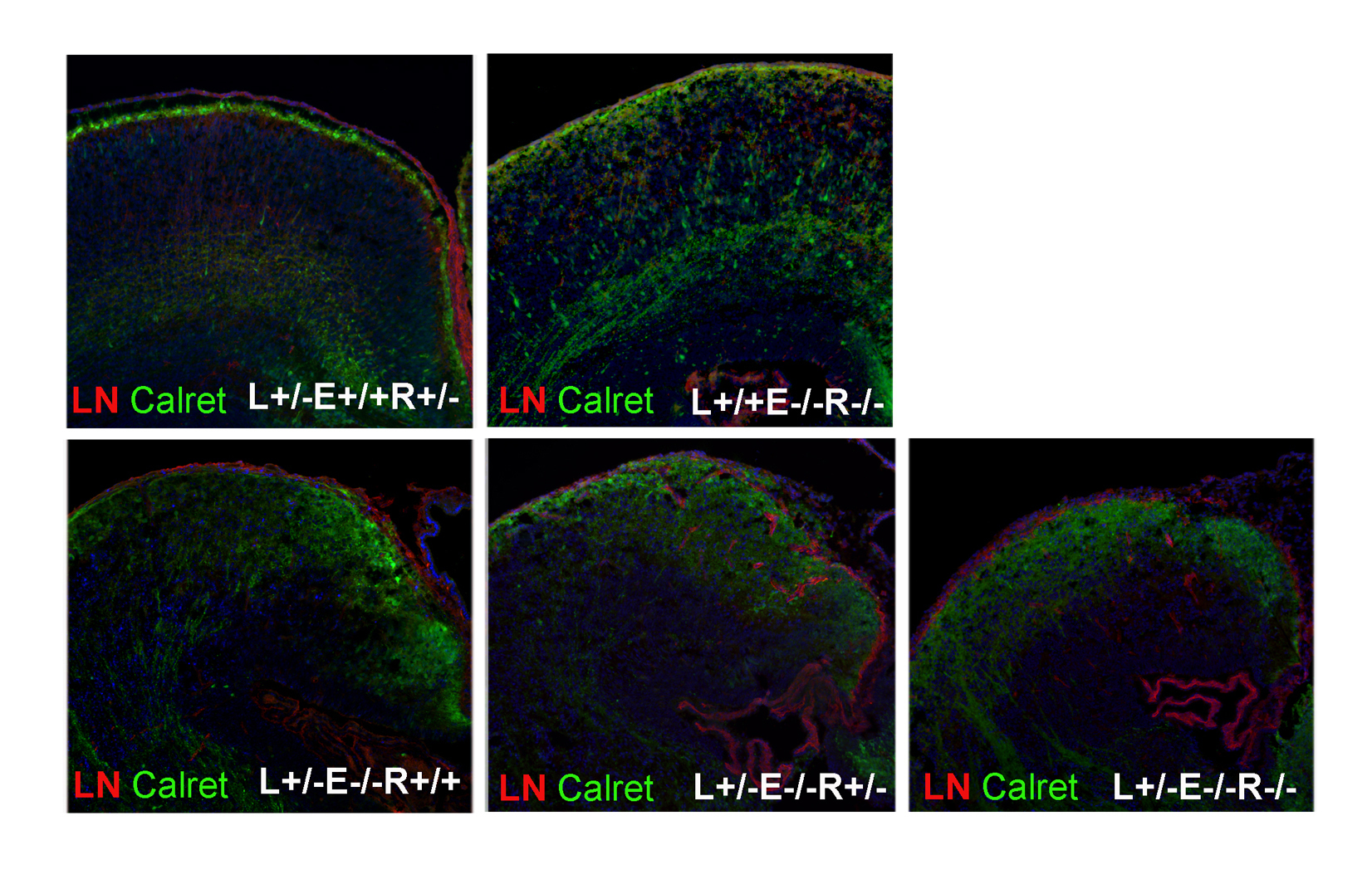

Supplement: Figure S9 — The Reelin independence of BM fragmentations in the Lis1+/−Nde1−/− mutants. Brains of Lis1+/−Nde1−/−Reln+/+, Lis1+/−Nde1−/−Reln+/−, and Lis1+/−Nde1−/−Reln−/− mice were analyzed immunohistologically with antibodies to laminin to highlight the BM (in red) and Calretinin to label C-R cells (in green). BM fragmentations and C-R cell ectopia were observed in all three mutants despite the fact that they expressed different levels of Reelin. Thus, the RGC basal-lateral morphology defect caused by in the Nde1−/−Lis1+/− mutation was not due to the elevated Reelin in the mutant cortex. (TIF) [file pbio.1001172.s009.tif]
